# Improving immunotherapy responses by dual inhibition of macrophage migration inhibitory factor and PD-1

**DOI:** 10.1172/jci.insight.191539

**Published:** 2025-10-22

**Authors:** Thuy T. Tran, Gabriela Athziri Sánchez-Zuno, Lais Osmani, Jasmine Caulfield, Caroline Naomi Valdez, Marta Piecychna, Lin Leng, Michelle E. Armstrong, Seamas C. Donnelly, Carlo B. Bifulco, Terri Clister, Rajan P. Kulkarni, Lin Zhang, Mario Sznol, Lucia Jilaveanu, Harriet M. Kluger, Insoo Kang, Richard Bucala

**Affiliations:** 1 Yale Cancer Center, Department of Internal Medicine, and; 2 Section of Rheumatology, Allergy & Immunology, Department of Internal Medicine, Yale School of Medicine, New Haven, Connecticut, USA.; 3 Department of Medicine, Trinity College Dublin, Dublin, Ireland.; 4 Earle A. Chiles Research Institute, Providence Cancer Institute, Portland, Oregon, USA.; 5 Department of Pathology, Yale School of Medicine, New Haven, Connecticut, USA.; 6 Department of Dermatology, Oregon Health & Science University, Portland, Oregon, USA.

**Keywords:** Immunology, Oncology, Cancer immunotherapy, Cytokines, Macrophages

## Abstract

Macrophage migration inhibitory factor (MIF) is an upstream regulatory cytokine that is associated with advanced disease and poor outcomes in multiple cancer types, including melanoma. We investigated whether anti-MIF therapy could enhance the antitumor effects of the immune checkpoint inhibitor anti–programmed cell death 1 (anti–PD-1) in 2 murine tumor models. The therapeutic efficacy of anti-MIF, alone or combined with anti–PD-1, was tested in the YUMMER1.7 melanoma and MC38 colorectal cancer models. Tumor growth and survival were assessed in untreated *Mif*-knockout (KO) and low-expression human MIF allele (CATT_5_) mice and compared with wild-type (WT) or high-expression MIF allele (CATT_7_) mice. Tumor-bearing animals underwent cytokine profiling, tumor immunohistochemistry, flow cytometry, and scRNA-Seq. We also correlated functional variant MIF alleles with melanoma incidence and progression in patients. Our results showed that combined anti-MIF and anti–PD-1 significantly reduced tumor growth, improved survival, and promoted tumor regression, accompanied by enhanced T_H_1 cytokine levels, increased macrophage activation–related cytokines, and increased type 1 conventional dendritic cells. scRNA-Seq analysis revealed an expansion of intratumor *Cd74/C1q/Aif1*-expressing macrophages, which exhibited an antitumor phenotype, in response to anti-MIF therapy. MIF-KO and CATT_5_ mice exhibited reduced tumor burdens compared with WT or CATT_7_ mice alone and in the presence of anti–PD-1. In patients with melanoma, the high-MIF expression genotype (-173C/C) occurred at higher frequencies compared with healthy controls. These findings highlight that the addition of anti-MIF to anti–PD-1 reduces tumor growth, enhances antitumor responses, prolongs survival, and augments key intratumor immune cell populations involved in immune activation against tumors. This approach merits further consideration for clinical trial development.

## Abstract 2

Combining anti-MIF with anti-PD-1 therapy enhances immune activation, reduces tumor growth, and improves survival in melanoma, highlighting potential for clinical application.

## Introduction

Skin cancer is the most prevalent malignancy in the United States, and melanoma accounts for the highest number of fatalities. Over 9,000 individuals die annually (1). The majority of melanomas result from ultraviolet sun exposure, but they may develop in non-sun-exposed areas, such as in acral lentiginous, mucosal, and uveal melanomas (2). Melanomas are well known to arise via immune evasion by inducing a local immune-suppressed tumor microenvironment (TME) (3).

Immunotherapy has shifted the paradigm of cancer treatment, but significant limitations in response and cure remain. Current immunotherapeutic strategies include immune checkpoint inhibition, cytokines, intratumor injections, or cellular based therapies (4). Despite the promise of these approaches for improved and durable clinical responses, nearly half of patients treated with immune checkpoint inhibitors (ICIs), for instance, do not benefit due to primary or secondary resistance (5). The need to develop new therapies to overcome ICI resistance remains, with efforts focusing on identifying superior or synergistic targets that overcome treatment resistance. Melanoma models have proven valuable for investigating the biology of ICI resistance and offer preclinical validation for novel therapies. Cytokine manipulation has reemerged as a tractable approach in oncology given the important role of these mediators in regulating immunity.

Macrophage migration inhibitory factor (MIF) was first described in 1966 as a product of T lymphocytes that inhibits the random migration of macrophages (6). MIF is now widely recognized as a pleiotropic cytokine secreted by multiple cell types to modulate the inflammatory and immune response (6). Additionally, MIF has direct pro-tumorigenic properties that include augmenting cell cycle progression, angiogenesis, and promoting oncogenesis by inhibiting the p53 and Rb tumor suppressors. MIF also enhances pathways involving cathepsin Z, IL-8, VEGF, MMP-9 and MMP-13, and CD44 (7).

Many of MIF’s pro-tumorigenic effects have been linked to the activation of its cognate receptor, CD74, which triggers sustained ERK1/2 phosphorylation and NF-κB activation to influence mitochondrial dynamics and inhibit apoptosis (8). Increased MIF expression has been correlated clinically with aggressive disease or increased tumor angiogenesis in patients with melanoma (9), glioblastomas (10), non–small cell lung cancer, and head and neck cancer (11, 12).

Previous research has demonstrated that inhibition of MIF/CD74 signaling in combination with ipilimumab, which targets the T cell coinhibitory signal, cytotoxic T lymphocyte antigen 4, can enhance cytotoxic CD8^+^ T cell infiltration and promote the conversion of macrophages to an M1-like phenotype within the TME (13). In this study, we evaluated if the combination of anti-MIF and anti–programmed cell death 1 (anti–PD-1) therapies could synergistically reduce tumor progression in the immune-responsive YUMMER1.7 melanoma and the MC38 colorectal carcinoma murine models. Our findings provide insights into the potential benefits of this combined immunotherapy approach for enhancing antitumor immune responses and improving therapeutic outcomes.

## Results

### Targeting both MIF and PD-1 improves antitumor responses.

**Figure 1:**
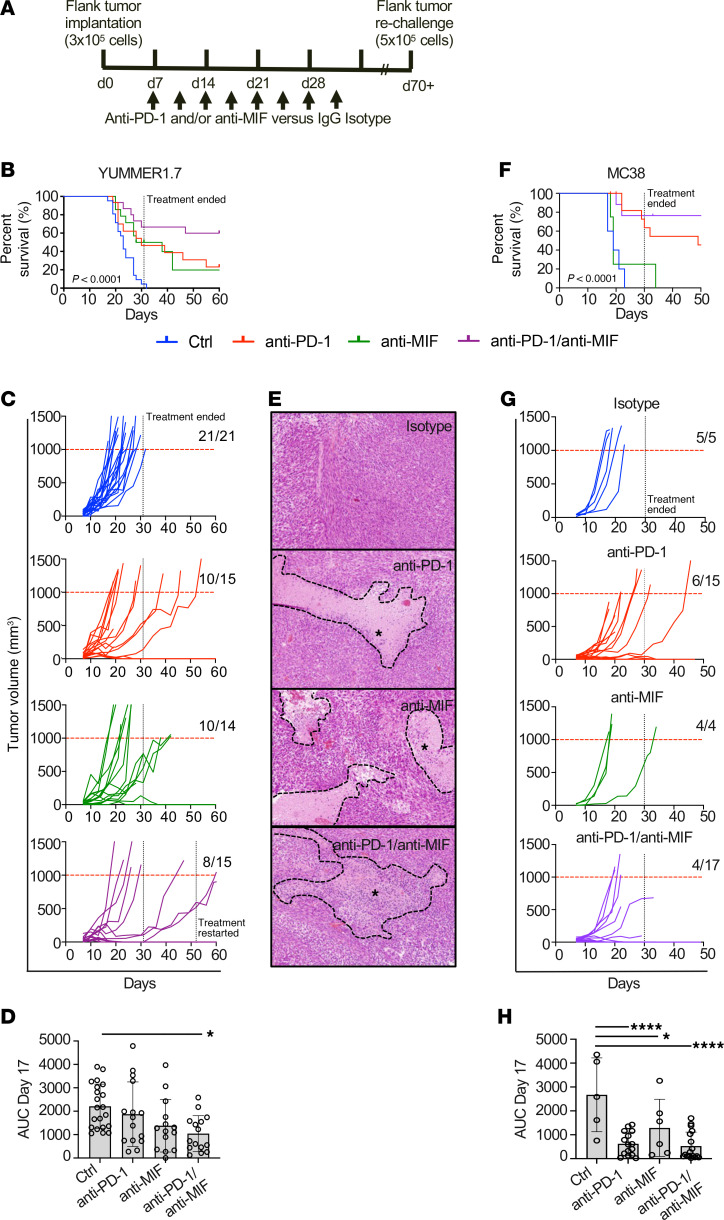
Anti–PD-1/anti-MIF improves survival and tumor responses. (**A**) Mice were randomized 7 days after tumor implantation and treated with anti–PD-1, anti-MIF, both antibodies, or isotype control (ctrl). Tumor volume and survival were monitored. (**B** and **C**) Kaplan-Meier survival analysis (log-rank test) and individual YUMMER1.7 tumor volumes over time. (**D**) Area under the curve (AUC) analysis (1-way ANOVA with Bonferroni correction) confirmed reduced tumor burden with combination therapy; endpoint was tumor volume > 1,000 mm³. (**E**) H&E staining (10× original magnification) of YUMMER1.7 tumors showed increased necrosis (dashed lines) and lymphocytic infiltration (*) with dual treatment. (**F** and **G**) Kaplan-Meier survival curves (log-rank test) showed improved survival and reduced tumor growth in the MC38 model with dual therapy. (**H**) By day 17, MC38 tumor growth was delayed in all treatment groups versus ctrl (1-way ANOVA with Bonferroni correction). Bars indicate SEM; circles show individual data points. Statistical significance is denoted as *P < 0.05, ****P < 0.0001.

We first assessed anti-MIF and anti–PD-1 antibody treatments in C57BL/6J mice injected subcutaneously with YUMMER1.7 melanoma cells. As expected, prolonged survival was observed for YUMMER1.7 tumor–bearing animals receiving PD-1 inhibition compared with the control (ctrl/isotype) group (*P* = 0.0025). MIF inhibition produced similar results to anti–PD-1 (*P* = 0.001 for ctrl versus anti-MIF, and *P* = 0.89 for anti–PD-1 versus anti-MIF). The dual-inhibition treatment (anti–PD-1/anti-MIF) compared with anti–PD-1 monotherapy or the control (ctrl/isotype) group resulted in superior survival (*P* = 0.08 and *P* < 0.0001, respectively) (Figure 1, A and B). Given that most isotype-treated animals were sacrificed by day 31 because of tumor growth >1,000 mm^3^, treatment was stopped to assess the impact on tumor growth in the absence of drug. Notably, 30% (3/10) of animals with initial response to anti–PD-1/anti-MIF had regrowth after treatment cessation while 70% (7/10) of these mice remained in response (Figure 1C). An analysis of the area under the curve (AUC) for tumor growth at day 17 revealed a significant reduction with dual anti–PD-1/anti-MIF treatment compared with the ctrl group (*P* = 0.01) (Figure 1D). Representative H&E stains of tumors highlighted areas of necrosis and immune infiltration (Figure 1E). Similar survival outcomes were observed in the colorectal MC38 model (Figure 1F), with most mice treated with anti–PD-1/anti-MIF exhibiting superior tumor rejection (Figure 1G). The AUC analysis at day 17 demonstrated a significant reduction in tumor size with dual anti–PD-1/anti-MIF treatment as well as anti–PD-1 and anti-MIF monotherapy when compared with the ctrl group (*P* < 0.001, *P* < 0.001, and *P* = 0.0176, respectively) (Figure 1H).To evaluate the presence of tumor immune memory in the YUMMER1.7 model, we rechallenged animals that had a complete rejection of tumor with a larger tumor load and observed the animals without treatment (Supplemental Figure 1A; supplemental material available online with this article; https://doi.org/10.1172/jci.insight.191539DS1). All animals had rapid rejection of their tumors. Animals then were challenged via intracardiac injection of tumor cells to model metastatic dissemination. A robust and prolonged antitumor immune response was evident (Supplemental Figure 1B).

**Figure 2:**
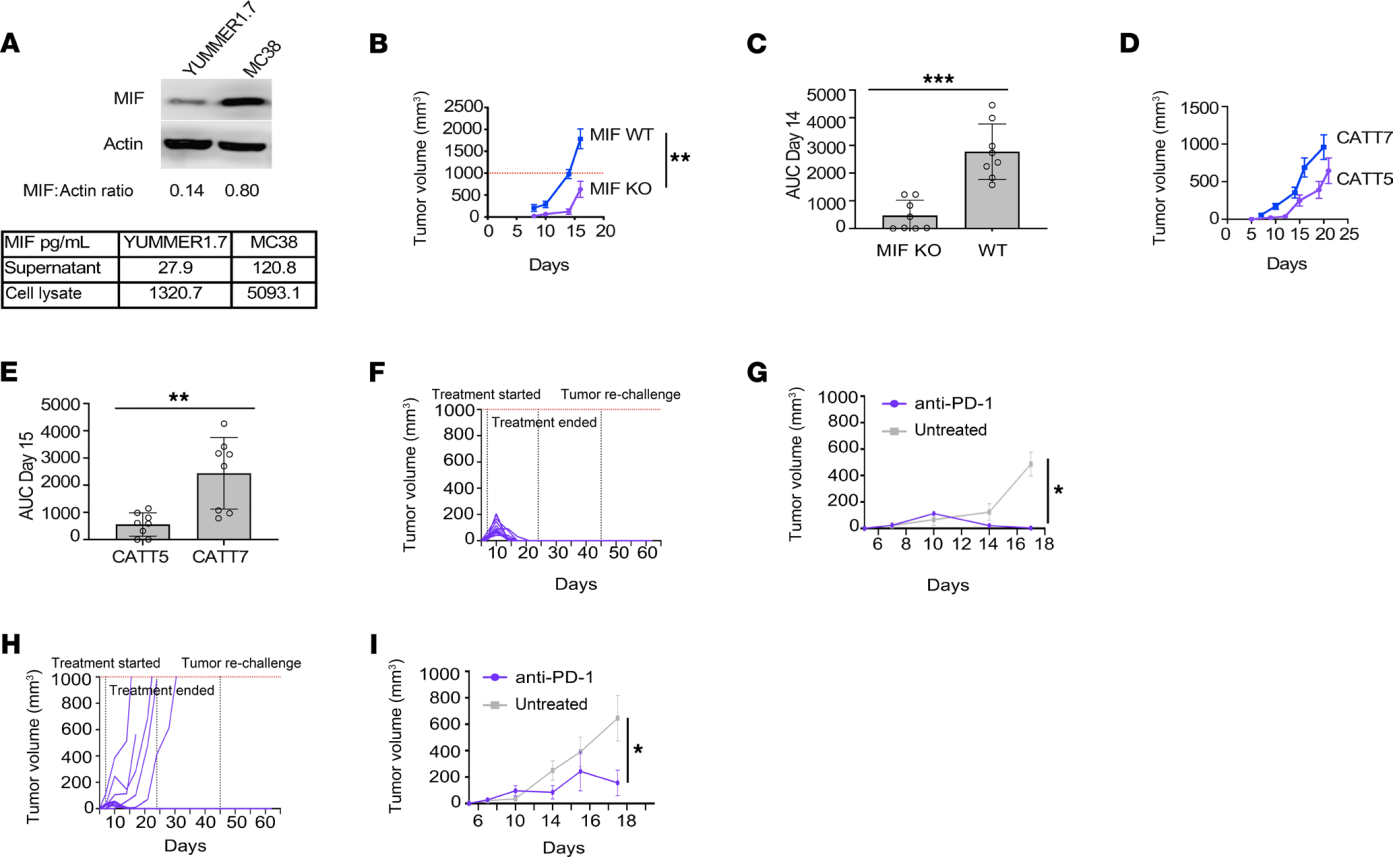
Reduced MIF expression limits tumor growth and enhances anti–PD-1 efficacy. (**A**) MIF protein levels were higher in MC38 than YUMMER1.7 cells by Western blot (MIF:Actin ratio) and by ELISA of supernatant and intracellular protein (MIF levels by pg/mL when 20,000 pg of total protein was added) (unpaired *t* test). (**B** and **C**) YUMMER1.7 tumors grew slower in MIF-knockout (KO) mice compared with WT controls, confirmed by growth curves (1-way ANOVA) and AUC analysis (unpaired *t* test) at day 14. (**D** and **E**) Tumors grew slower in low-MIF CATT_5_ mice versus high-MIF CATT_7_ mice, with significantly reduced tumor burden by AUC at day 15 (unpaired *t* test, *P* < 0.001). (**F** and **G**) Anti–PD-1 treatment further reduced YUMMER1.7 tumor growth in MIF-KO mice (unpaired *t* test). (**H** and **I**) Similar anti–PD-1 effects were seen in CATT_5_ mice (unpaired *t* test). Bars indicate SEM; circles show individual data points. Statistical significance is denoted as *P < 0.05, **P < 0.01, ***P < 0.001.

The YUMMER1.7 and MC38 tumors are established models for studying sensitivity to ICIs. We next assessed the effect of dual treatment in the widely utilized but less immune-sensitive B16F10 melanoma cells, which differ from YUMMER1.7 in lacking the typical oncogenic mutations and diversity of DNA mutations associated with human melanoma (14). No difference in tumor progression or percentage survival was observed between the different treatments in this model, consistent with B16F10 representing a more immunologically “cold” tumor (*P* > 0.05) (Supplemental Figure 2A). MIF is expressed by both tumor and immune cells (15), and high MIF levels are found within and secreted from YUMMER1.7 and MC38 cells (Figure 2A). Given the potential of tumor- or host-derived MIF in affecting tumor growth and the immune response, we assessed tumor growth in MIF gene–knockout (*Mif*^–/–^ or MIF-KO) mice. YUMMER1.7 tumor growth was significantly delayed in the MIF-KO group compared with WT controls (*P* = 0.0095) (Figure 2B). Furthermore, AUC analysis at day 14 indicated a marked reduction in overall tumor burden in MIF-KO animals compared with WT (*P* = 0.0002) (Figure 2C).

Human *MIF* transcription is regulated by a 4-nucleotide promoter microsatellite (-794 CATT_5-8_), with higher CATT repetition associated with increased *MIF* mRNA expression and MIF protein production (16). To evaluate the potential impact on tumorigenesis of this natural genetic variation in human *MIF* expression, we examined YUMMER1.7 tumor growth in mice engineered to express the commonly occurring -794 CATT_5_ low-expression or -794 CATT_7_ high-expression *MIF* alleles in place of endogenous murine *Mif* (17). Notably, the low genotypic human *MIF* expresser mice showed a similar reduction in tumor burden compared to the high genotypic human *MIF* expresser mice, mirroring results in the MIF-KO animals. CATT_5_ mice had a delay in tumor establishment of about 1 week (*P* = 0.0047) and a reduction in tumor volume by AUC analysis at day 15 when compared with the high genotypic human *MIF* expresser mice (*P* < 0.001) (Figure 2, D and E). To assess for differences in tumor versus host TME MIF expression in regulating tumor growth, MIF-deficient YUMMER1.7 cells were generated from a diploid line (Supplemental Figure 3, A and B). YUMMER1.7 diploid *Mif*^–/–^ tumors grew similarly to parental tumors in MIF WT mice (Supplemental Figure 3C), thus implicating a more important role for MIF in the TME specifically in this model.

When MIF-KO mice with YUMMER1.7 tumors were treated with anti–PD-1, 100% of the animals (*n* = 5 mice injected bilaterally with tumors) had rapid and complete regression of tumors by day 20 and ongoing antitumor responses after cessation of anti–PD-1 therapy and upon tumor rechallenge, which was significantly improved compared with untreated MIF-KO mice (Figure 2, F and G). Similarly, when CATT_5_ mice with YUMMER1.7 tumors were treated with anti–PD-1, 3/5 mice had rapid tumor growth. Two of 5 mice had complete regression of their tumors; these animals had ongoing antitumor responses after the cessation of anti–PD-1 therapy and upon later tumor rechallenge, which also was significantly improved compared with untreated CATT_7_ tumors (Figure 2, H and I).

### Anti–PD-1 and anti-MIF combination therapy augments T_H_1 responses and macrophage activation.

**Figure 3:**
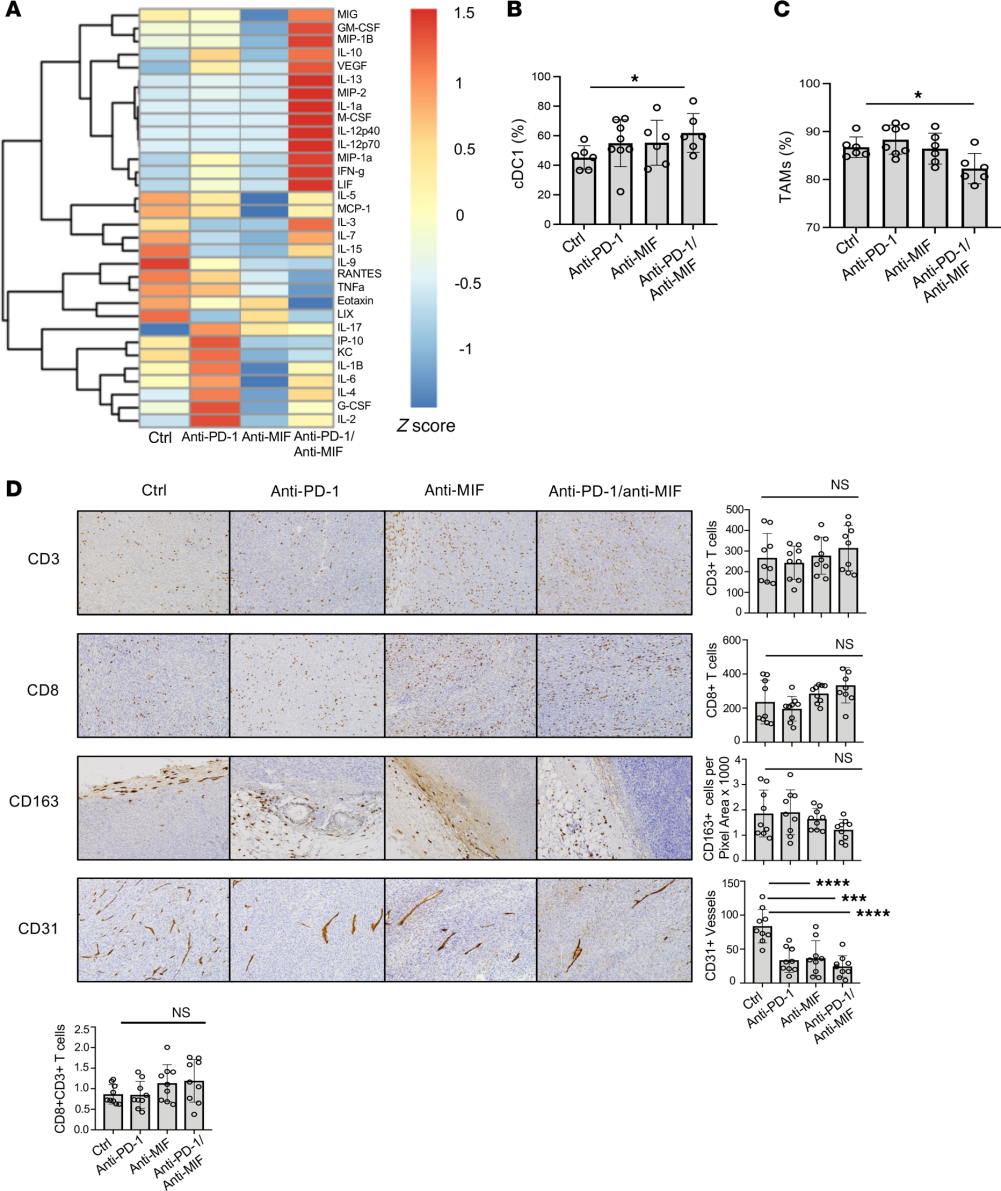
Dual inhibition of PD-1 and MIF enhances cytokines/chemokines associated with T_H_1 and macrophage activation, increases cDC1s, decreases TAMs, and decreases tumor-associated angiogenesis. (**A**) Plasma from tumor-bearing mice prior to and after 2 doses of treatment was evaluated (*n* = 5 per group). Anti–PD-1/anti-MIF treatment enhanced T_H_1 signaling, as evidenced by increased markers of T cell stimulation (MIG and IL-1α), particularly T_H_1 cytokines (GM-CSF, IL-12p40, IL-12p70, and IFN-γ), and markers of macrophage activation (MIP-1β, MIP-2, M-CSF, and MIP-1α). (**B**) Flow cytometry analysis revealed an increase in the cDC1 population (CD45^+^Ly6C^–^CD3^–^CD19^–^TCR^–^CD11c^+^MHCII^+^CD172^lo^XCR1^hi^) and a (**C**) decrease in tumor-associated macrophages (TAMs) (F4/80^hi^MHCII^hi^CD64^hi^) within YUMMER1.7 tumors upon combined anti–PD-1/anti-MIF therapy compared with ctrl (1-way ANOVA with Bonferroni’s correction). (**D**) Representative photos of YUMMER1.7 tumors stained with anti-CD3, anti-CD8, anti-CD163, and anti-CD31. Photos taken on 10× original magnification. Treated mice showed significantly lower CD31^+^ vessel quantity compared with the control group. There was a nonsignificant trend in increased CD8^+^ T cells upon anti–PD-1/anti-MIF therapy (*P* = 0.08, 1-way ANOVA with Bonferroni correction). Statistical significance is denoted as *P < 0.05, ***P < 0.001, and ****P < 0.0001.

To understand the mechanisms underlying anti–PD-1/anti-MIF–induced tumor regression, we first measured representative circulating cytokines and chemokines in plasma collected from YUMMER1.7 tumor–bearing mice prior to and after 2 doses of treatment. When compared with monotherapy, dual therapy was associated with increased T_H_1 type cytokines (e.g., GM-CSF, IL-12p40, IL-12p70, and IFN-γ) and the T cell–stimulatory cytokines MIG (CXCL9) and IL-1α. Several cytokines and chemokines indicative of macrophage activation (e.g., macrophage inflammatory protein 1β [MIP-1β], MIP-2, M-CSF, and MIP-1α) also were increased by dual therapy when compared with anti–PD-1, anti-MIF, or isotype treatment (Figure 3A).

### Anti–PD-1 and anti-MIF combination therapy increases tumor infiltration of type 1 conventional dendritic cells while decreasing tumor-associated macrophages and angiogenesis.

We next evaluated YUMMER1.7 tumor-infiltrating immune cell types by flow cytometry after the different antibody treatments. We did not observe any differences in general immune populations, including in T cells and myeloid-derived suppressor cells (MDSCs), between treatment groups (Supplemental Figure 4, A–F). There were an increase in the percentage of type 1 conventional dendritic cells (cDC1s: CD45^+^Ly6C^–^CD3^–^CD19^–^TCR^–^CD11c^+^MHCII^+^CD172^lo^XCR^hi^) (*P* = 0.047) and a corresponding decrease in tumor-associated macrophages (TAMs) (F4/80^hi^MHCII^hi^CD64^hi^) within YUMMER1.7 tumors after combination anti–PD-1/anti-MIF therapy when compared with the ctrl group (*P* = 0.039) (Figure 3, B and C). There were no statistical differences in these cell types among the monotherapy treatment groups.

We additionally evaluated the effects of combined PD-1/MIF inhibition by immunohistochemistry of YUMMER1.7 tumors collected after survival analyses in the different treatment groups (Figure 3D). Tumors from anti–PD-1/anti-MIF–treated mice showed a trend toward more CD3^+^ and CD8^+^ T cells, fewer CD163^+^ (M2) macrophages, and higher CD8/CD3 ratios that did not reach statistical significance. Mice treated with mono- or dual therapy, however, did exhibit a significantly lower quantity of CD31^+^ blood vessels compared with the ctrl group (anti–PD-1 *P* < 0.0001, anti-MIF *P* < 0.001, anti–PD-1/anti-MIF *P* < 0.0001), which is in agreement with MIF’s previously reported action in promoting tumor angiogenesis (18).

### scRNA-Seq analysis reveals an expansion of intratumor Cd74/C1q/allograft inflammatory factor 1–expressing macrophages in response to anti-MIF therapy.

**Figure 4:**
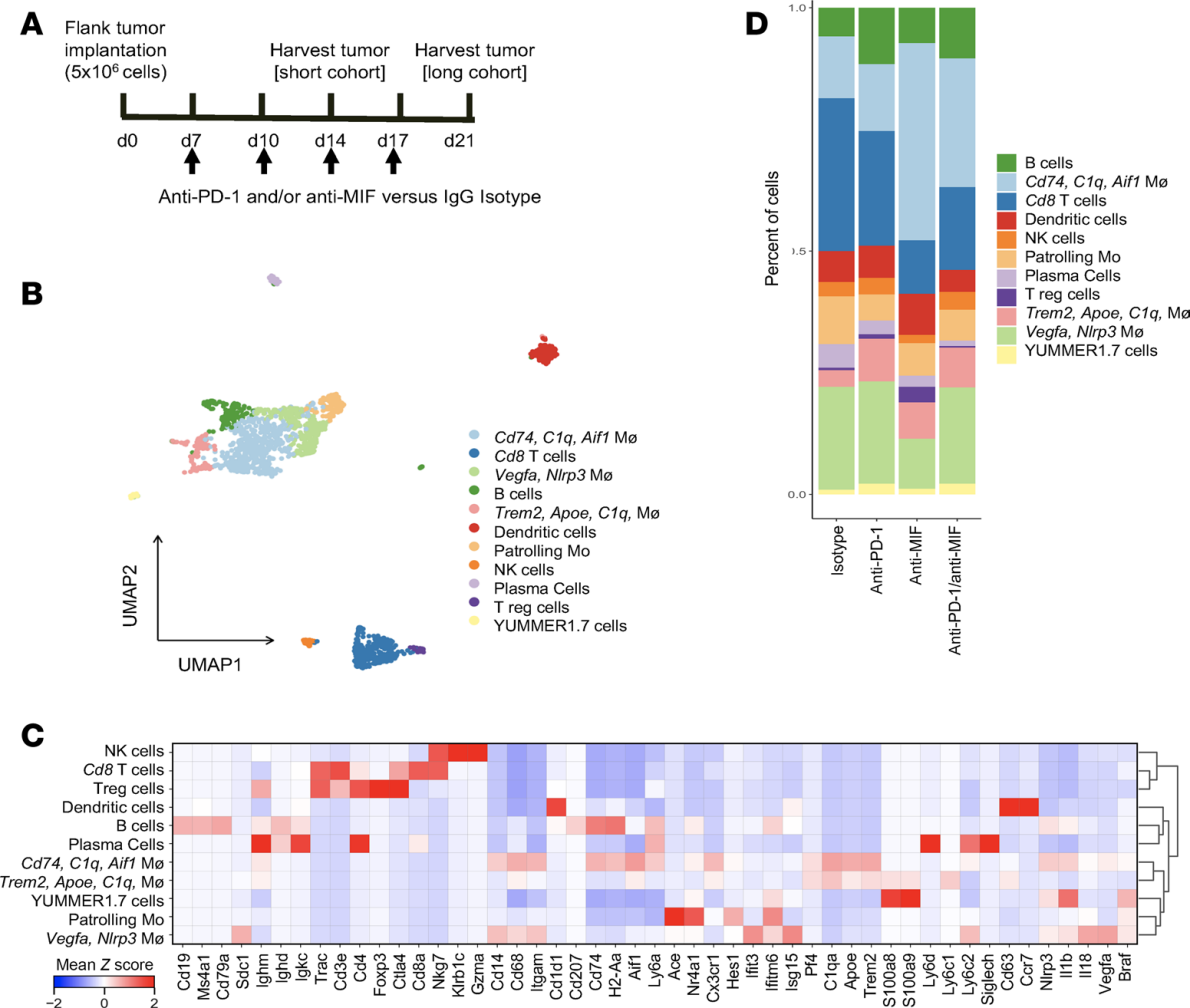
Anti-MIF therapy reshapes myeloid cell diversity within the TME. (**A**) Scheme of YUMMER1.7 tumor injection and treatment for single-cell RNA-Seq (scRNA-Seq) analysis, including endpoints for the short treatment (2 doses, day 14) and long treatment cohorts (4 doses, day 21). (**B**) Uniform manifold approximation and projection (UMAP) analysis with nonbiased clustering into 11 cell types from the long treatment cohort. Mo, monocytes; Mø, macrophages. (**C**) Scaled heatmap of select differentially expressed genes (DEGs) and canonical markers used for cluster annotation. *Aif1*, allograft inflammatory factor 1. (**D**) Stacked bar plot of cluster proportions across the different treatment groups. Myeloid cells represent a majority of immune cells within the TME.

To better investigate the effect of anti-MIF on the TME, we subcutaneously injected YUMMER1.7 melanoma cells into mice and treated them for either 1 week (2 doses, short treatment) or 2 weeks (4 doses, long treatment) before isolating tumors for single-cell RNA-Seq (scRNA-Seq) analysis (Figure 4A). Unsupervised clustering identified 11 distinct cell populations across both treatment cohorts, with myeloid cells constituting the majority of immune cells within the TME (Figure 4, B–D). Our analysis focused on myeloid-derived populations, which overall showed consistent frequencies between short and long treatment groups (Supplemental Figure 5, A and B). Given this similarity, we will primarily refer to the long treatment cohort in subsequent discussions.

**Figure 5:**
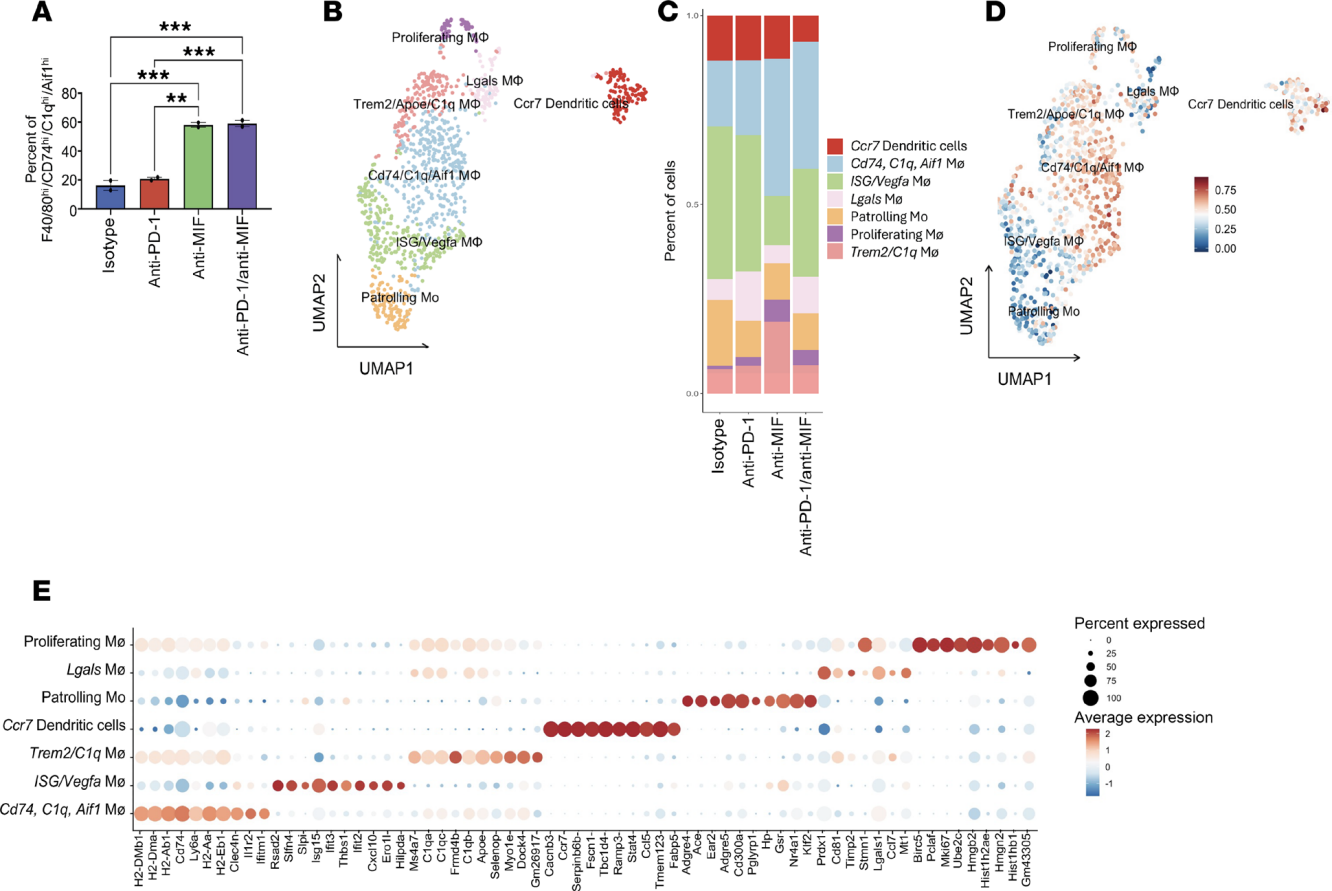
Expansion of *Cd74/C1q/Aif1*-expressing intratumoral macrophages in response to anti-MIF therapy. (**A**) Flow cytometry validation indicating a significant increase in intratumoral *Cd74/C1q/Aif1* macrophages with anti-MIF–containing therapies (1-way ANOVA with Bonferroni correction). (**B**) UMAP of myeloid subset reclustering identified 7 cell types. Further analysis revealed a diverse population of macrophages and dendritic cells. (**C**) Stacked bar plot of myeloid cluster proportions across the different treatment groups in the long treatment cohort. (**D**) Feature plot of module scores highlighting myeloid cells with upregulation of genes associated with the Kyoto Encyclopedia of Genes and Genomes (KEGG) antigen processing and presentation gene set. (**E**) Dot plot of top DEGs for each myeloid cell type. Statistical significance is denoted as **P < 0.01, ***P < 0.001.

There was an expansion of *Cd74/C1q/Aif1*-expressing macrophages in the anti-MIF treatment groups (anti-MIF alone and anti–PD-1/anti-MIF) when compared with the anti–PD-1 monotherapy or isotype groups (Figure 4D). This finding was validated on flow cytometry analysis (Figure 5A and Supplemental Figure 6). Further subclustering analysis of the myeloid population revealed a diverse population of macrophages and dendritic cells (DCs) (Figure 5, B, C, and E) with consistent expansion of the *Cd74/C1q/Aif1*-expressing macrophages after anti-MIF–containing therapies (Figure 5C). *Cd74/C1q/Aif1* macrophages and *Ccr7* DCs (upregulated *Cd1d1/Ccr7/Cd63*) showed increased expression of genes within the Kyoto Encyclopedia of Genes and Genomes (KEGG) antigen processing and presentation gene set, which suggests these populations are professional antigen-presenting cells (Figure 5D). Additional flow cytometry analyses showed a nonsignificant trend toward increased cDC populations with anti-MIF but significant increases in cDCs expressing Cd74 and Ccr7 separately, compared with anti–PD-1 treatment, highlighting DC heterogeneity (*P* < 0.05). There was a nonsignificant trend in decreased MDSCs, which could be limited by the small sample size of available, matched tumors after processing for scRNA-Seq (Supplemental Figure 7).

### Cd74/C1q/Aif1-expressing macrophages exhibit enhanced antigen presentation and proliferation gene expression profiles in response to anti-MIF therapy.

**Figure 6:**
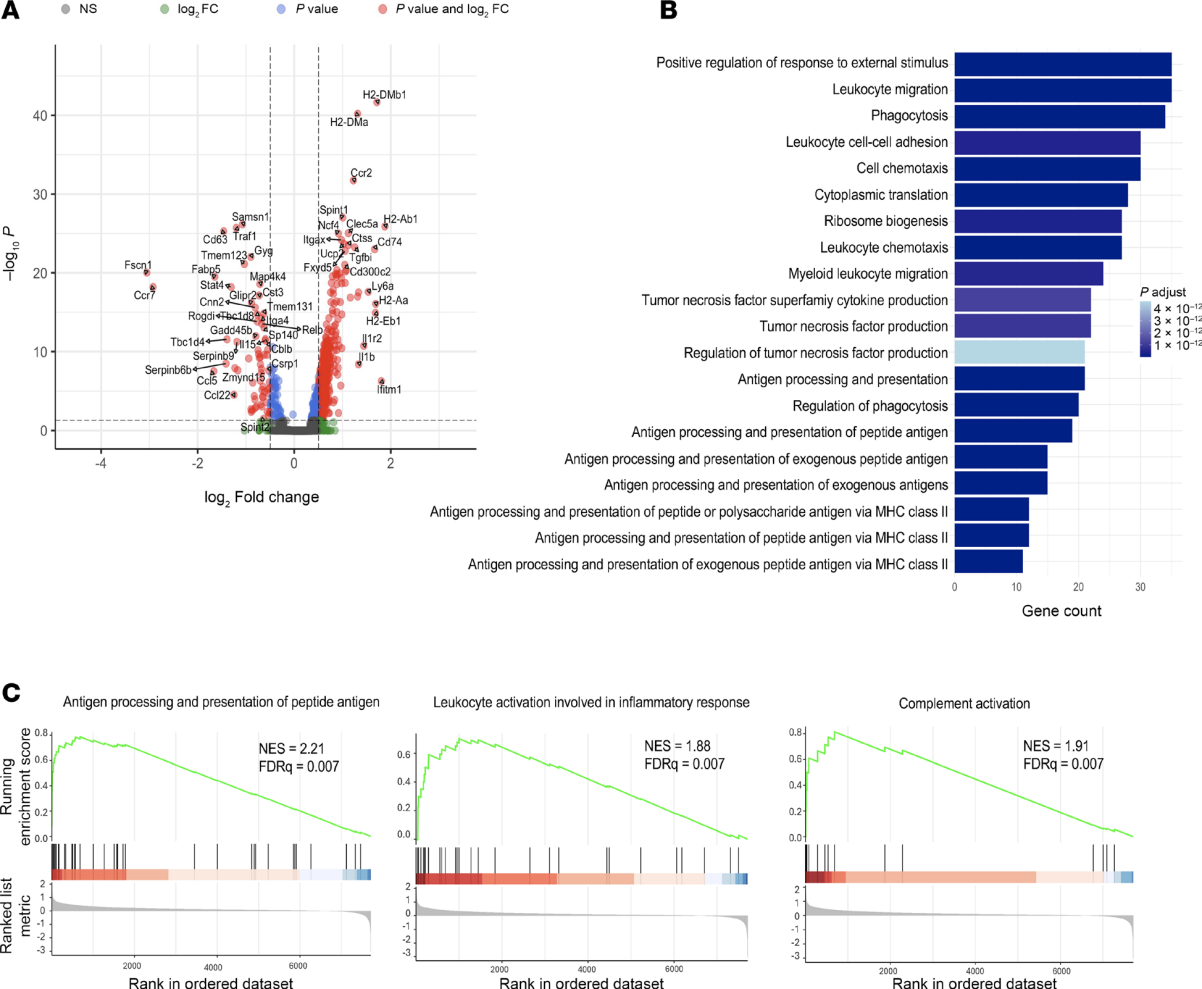
Anti-MIF therapy enhances gene expression associated with antigen presentation and phagocytosis of *Cd74/C1q/Aif1* macrophages. (**A**) Volcano plot of DEGs from *Cd74/C1q/Aif1*-expressing macrophages versus other myeloid cell clusters (adjusted *P* < 0.05, |log2fold-change| > 0.5). (**B**) Bar plot demonstrating top 20 enriched Gene Ontology Biological Process gene sets by overrepresentation analysis from upregulated DEGs in *Cd74/C1q/Aif1*-expressing macrophages. (**C**) Gene set enrichment analysis plots of antigen processing and presentation of peptide antigen, leukocyte activation involved in inflammatory response, and complement activation gene sets. NES, normalized enrichment score.

*Cd74/C1q/Aif1*-expressing macrophages are characterized by the expression of *Aif1*, which is vital for macrophage activation and phagocytosis (19). To further characterize this macrophage subpopulation, we analyzed our expression data for DEGs using the Wilcoxon rank-sum test (FDR < 0.05, |fold change| > 1.25). When compared with other myeloid-derived populations (Figure 6A), there was a significant upregulation of genes for MHC class II antigen presentation, phagocytosis, and cell migration (e.g., *H2-dm, Ctsb/l/s*, and *Ifi30*). We also identified significant upregulation of genes associated with *Tnf* synthesis and function (Figure 6, B and C).

**Figure 7:**
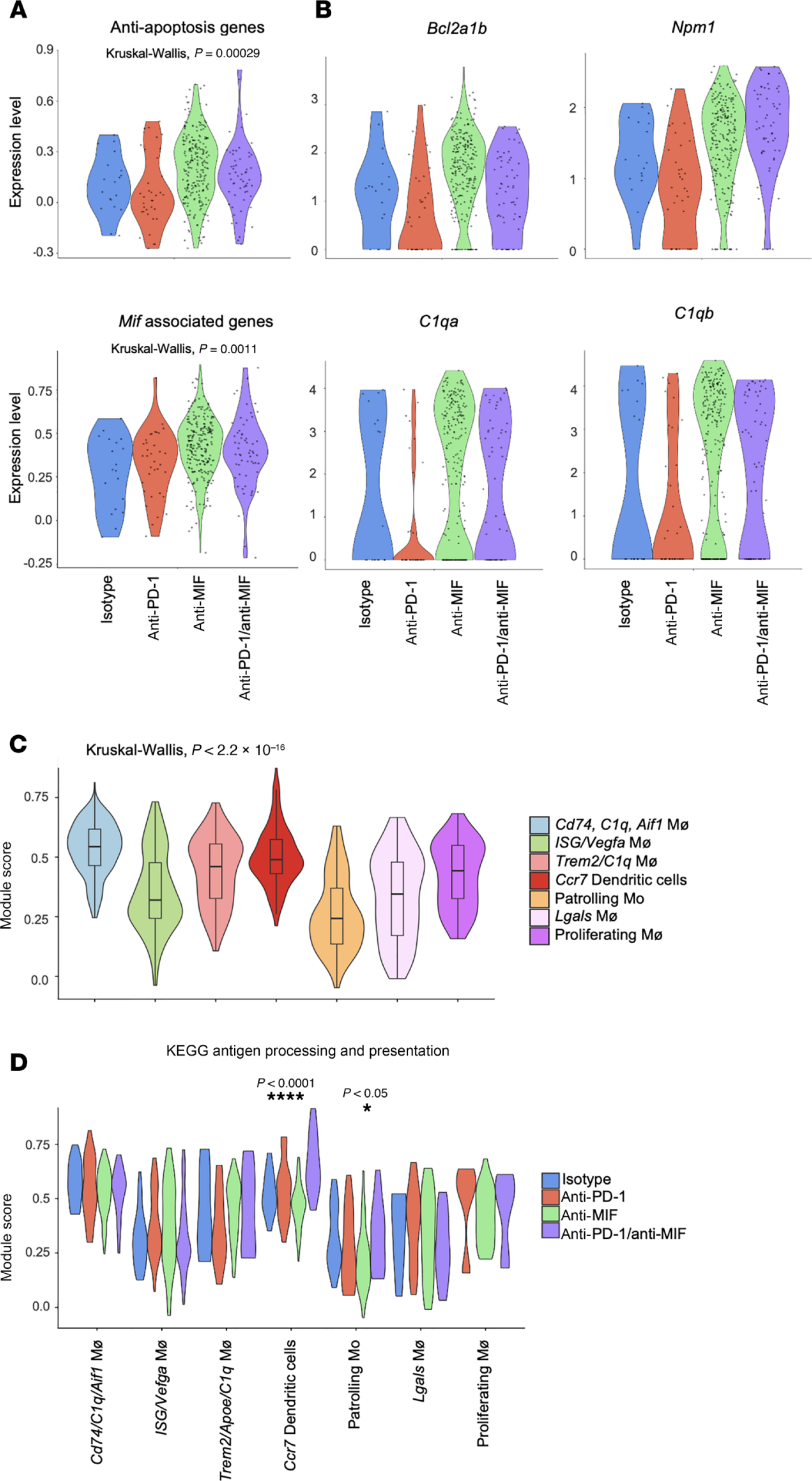
*Cd74/C1q/Aif1* macrophages exhibit pro-survival and antigen presentation signatures enhanced by anti-MIF therapy. (**A**) Violin plots of module scores for expression of antiapoptosis genes and *Mif* associated genes gene sets. Kruskal-Wallis test used for statistical comparison of module scores. (**B**) Violin plots of *Bcl2a1b*, *Npm1*, *C1qa*, and *C1qb* expression. (**C**) Violin plot of module scores for expression of the KEGG antigen processing and presentation gene set, demonstrating increased activity in *Cd74/C1q/Aif1* macrophages and *Ccr7* DCs. Kruskal-Wallis test used for statistical comparison of module scores. (**D**) Split violin plot of module scores for expression of the KEGG antigen processing and presentation gene set across the different treatment groups (Kruskal-Wallis: *P < 0.05; ****P < 0.0001).

Notably, the *Cd74/C1q/Aif1* macrophages demonstrated higher expression of *Mif*-associated genes as well as antiapoptosis genes across anti-MIF and anti–PD-1/anti-MIF treatment groups when compared with the anti–PD-1 monotherapy or ctrl groups (Figure 7A). This macrophage population also showed a significant increase in the expression of proliferation genes, including *Bcl2a1b* and *Npm1*, in the anti-MIF and anti–PD-1/anti-MIF treatment groups when compared with the anti–PD-1 monotherapy and ctrl groups. There also was a trend toward higher expression of the complement genes *C1qa* and *C1qb* in the anti-MIF and anti–PD-1/anti-MIF treatment groups when compared with the anti–PD-1 monotherapy and ctrl groups (Figure 7B). As expected from the initial DEG analysis (Figure 6, A and B), the *Cd74/C1q/Aif1* macrophages and DCs showed higher expression of antigen-processing and -presenting genes (Figure 7C). The *Ccr7* DCs from anti-MIF–treated mouse tumors also exhibited higher expression of antigen-processing and -presenting genes (Figure 7D).

**Figure 8:**
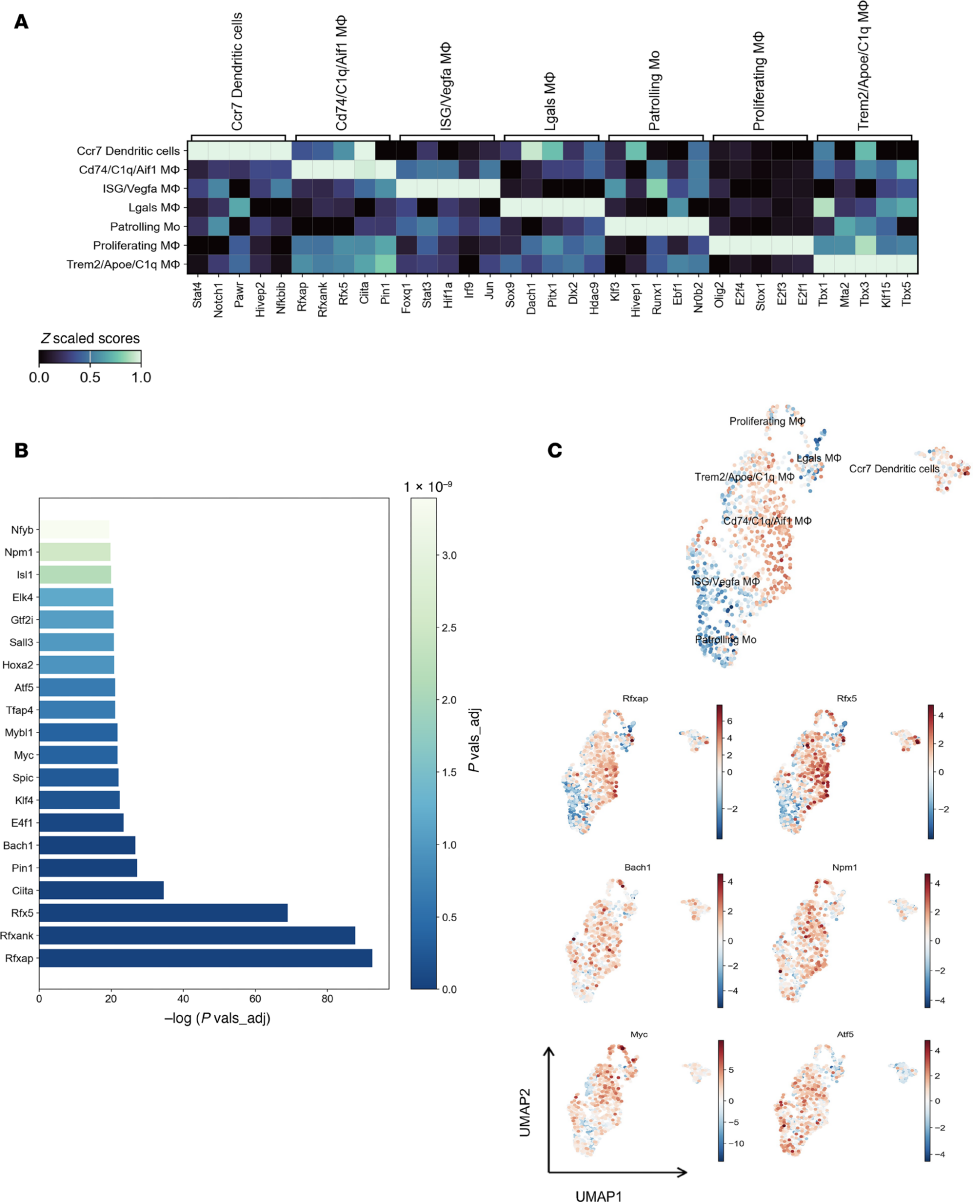
Transcription factor activity analysis of *Cd74/C1q/Aif1* macrophages. (**A**) Scaled heatmap of top 5 inferred transcription factor activities per myeloid cell cluster demonstrates increased activity of *Rfxap*, *Rfxank*, *Rfx5*, and *Ciita* transcription factors in *Cd74/C1q/Aif1* macrophages. (**B**) Bar plot of top 20 inferred transcription factor activities in *Cd74/C1q/Aif1* macrophages shows significant increases in *Npm1* and *Atf5* transcription factor activity, known to be associated with monocyte-to-macrophage differentiation. (**C**) Feature plots of inferred activities of select transcription factors.

We next investigated the transcription factor activities within the *Cd74/C1q/Aif1* macrophage population to gain deeper insights into their functional dynamics. Analysis of inferred transcription factor activity across various myeloid cell clusters revealed significant upregulation of *Rfxap*, *Rfxank*, *Rfx5*, and *Ciita* — key regulators of MHC class II expression and antigen presentation (20, 21) — reinforcing the enhanced antigen-presenting capabilities of this macrophage subset within the TME of the anti-MIF–treated mice (Figure 8A).

Further analysis of the top 20 active transcription factors within *Cd74/C1q/Aif1*-expressing macrophages showed a marked increase in *Npm1* and *Atf5* activity, which are crucial for promoting monocyte-to-macrophage differentiation via the NF-κB activation pathway (22, 23) (Figure 8B). Feature plots provided additional visualization of the activity levels of these transcription factors, highlighting their pivotal roles in shaping the functional profile of this macrophage population (Figure 8C).

### Anti–PD-1/anti-MIF combination therapy induced significant changes in immune cell interactions and signaling dynamics within the TME when compared with anti–PD-1 monotherapy.

**Figure 9:**
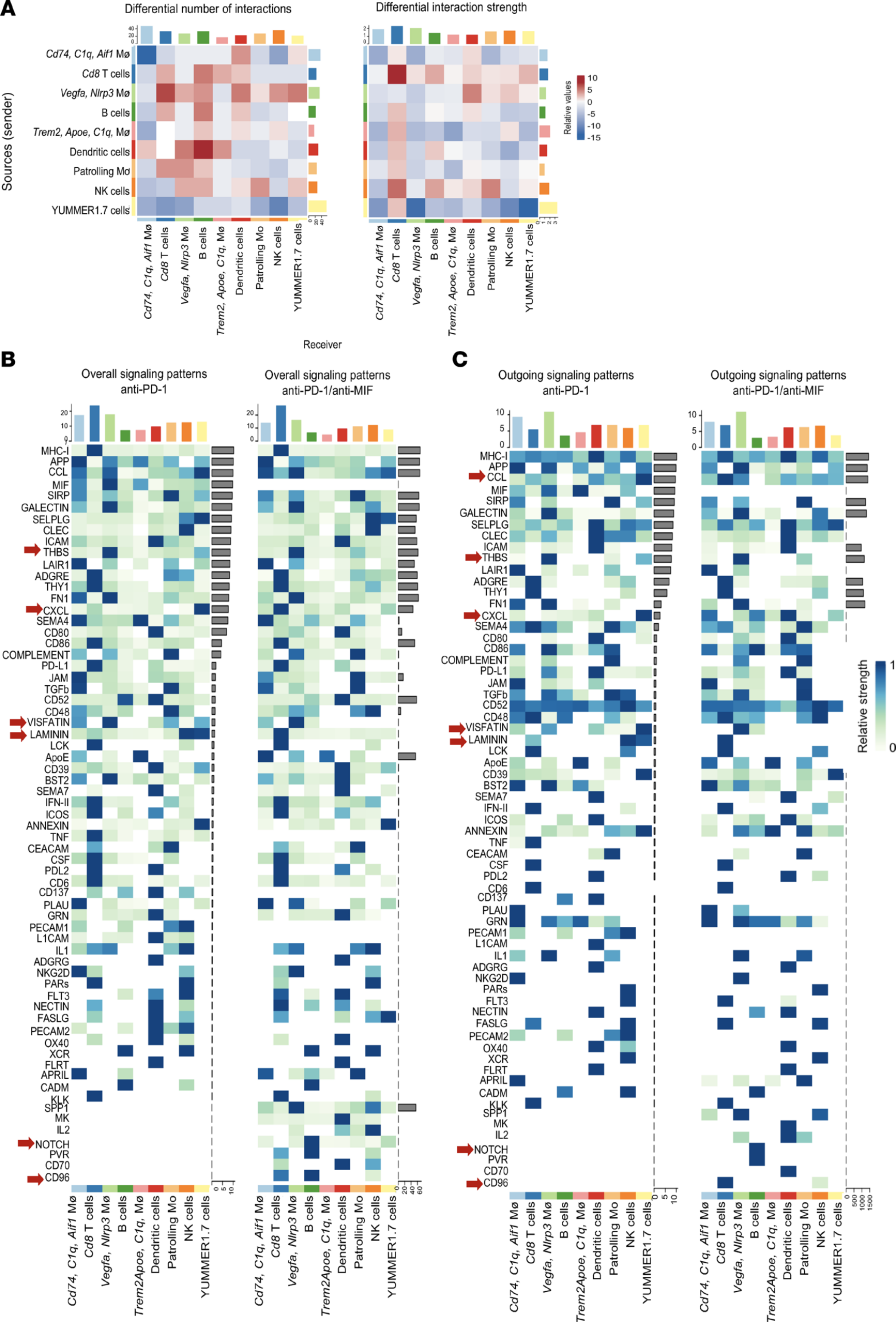
Dual anti–PD-1/anti-MIF therapy increases immune cell network connectivity in the TME. (**A**) Heatmaps demonstrating differential numbers and strengths of cell interactions between the anti–PD-1/anti-MIF treatment group relative to the anti–PD-1 treatment group (the bar plot at the top represents the sum of incoming signaling for each cell type, and the bar plot at the right represents the sum of outgoing signaling for each cell type). (**B** and **C**) Heatmaps of overall signaling patterns in both treatment conditions (colored bars represent sum of total signaling per cell type).

The combination of anti–PD-1 and anti-MIF therapy markedly altered immune cell interactions and signaling dynamics within the TME compared with anti–PD-1 monotherapy. Notably, there were an increase in interactions and strengthened connections among key immune cell populations in the anti-MIF–treated mice. *Cd74/C1q/Aif1* macrophages exhibited increased interactions with DCs and YUMMER1.7 cells and slightly strengthened interactions with CD8^+^ T cells and DCs. CD8^+^ T cells showed increased interactions with B cells, *Trem2/Apoe/C1q* macrophages, as well as DCs. They also showed increased strength of interactions with *Vegfa/Nlrp3* macrophages, B cells, patrolling Mo, YUMMER1.7 cells, NK cells, and DCs. Similarly, *Vegfa/Nlrp3* macrophages displayed increased interactions with CD8^+^ T cells, B cells, DCs, patrolling Mo, YUMMER1.7 cells, and NK cells. In contrast, tumor cells showed a marked reduction in both the number and strength of interactions after anti–PD-1/anti-MIF combination therapy, while selectively strengthening connections with CD8^+^ T cells (Figure 9A and Supplemental Figure 8A).

**Figure 10:**
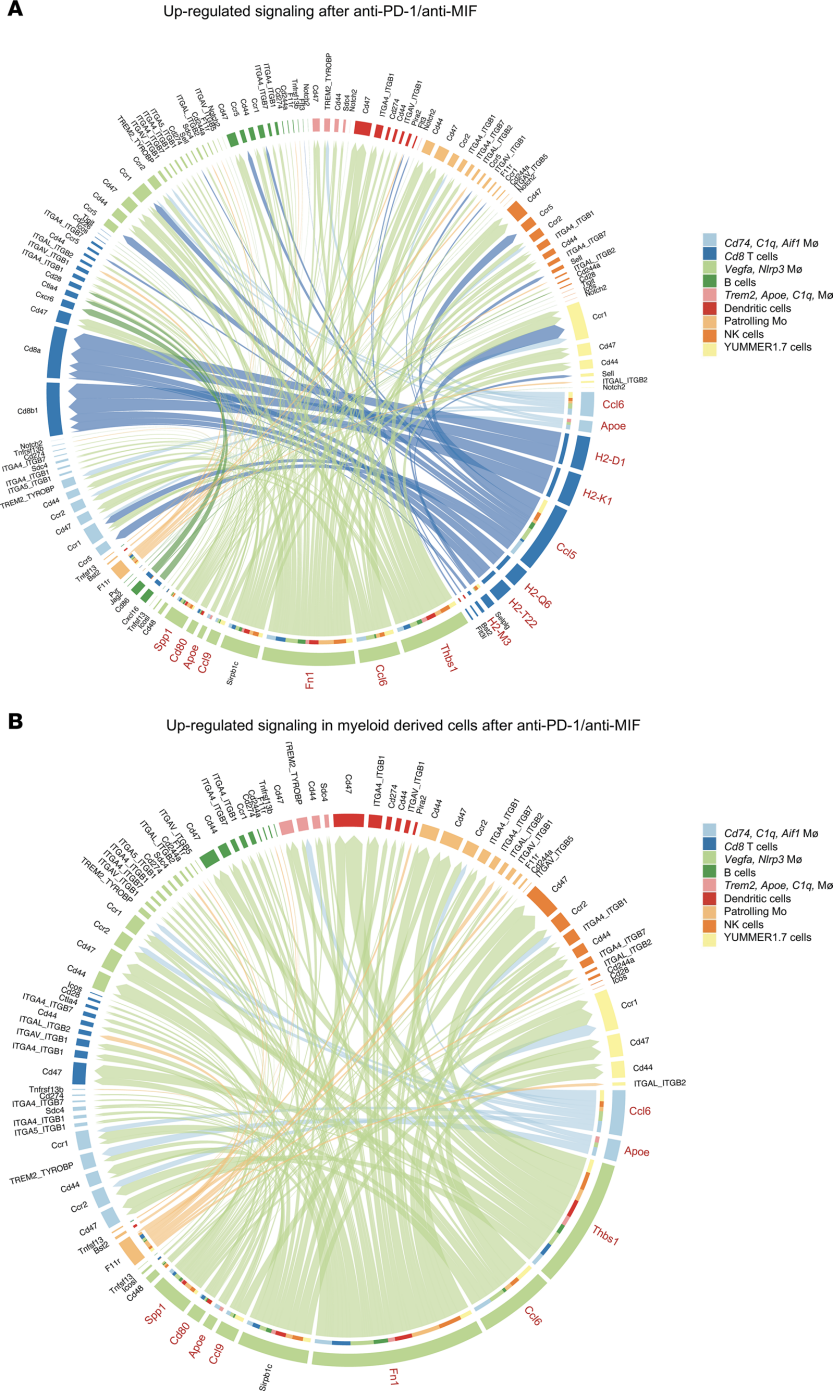
Enhanced antigen presentation and chemotactic signaling pathways upon combined anti–PD-1/anti-MIF therapy. (**A**) Chord diagram of upregulated ligand-receptor interactions in the anti–PD-1/anti-MIF treatment group relative to the anti–PD-1 treatment group. (**B**) Chord diagram of upregulated ligand-receptor interactions with myeloid cells as the source.

Cellular crosstalk analysis revealed significant changes in signaling patterns after anti–PD-1/anti-MIF therapy. Tumor cells exhibited reduced *Ccl* outgoing signaling strength and *Cxcl*, *Thbs*, *Visfatin*, and *Laminin* overall and outgoing signaling strength. Conversely, CD8^+^ T cells and *Vegfa/Nlrp3* macrophages demonstrated enhanced outgoing *Ccl* signaling. *Cxcl* outgoing signals were strengthened in *Cd74/C1q/Aif1* macrophages, B cells, and DCs. CD8^+^ T cells displayed increased *Cd96* signaling while B cells showed enhanced *Notch* signaling (Figure 9, B and C). Further analysis verified significant alterations in signaling pathways within the TME because of anti–PD-1/anti-MIF therapy. Enhanced outgoing MHC class I (*H2-K1*, *H2-D1*, *H2-Q6*, *H2-M3*, and *H2-T22*) signaling in CD8^+^ T cells indicated improved immune activation and antigen processing (Figure 10A). Notably, *Cd80* signaling from *Vegfa/Nlrp3* macrophages was heightened toward DCs, *Cd74/C1q/Aif1* macrophages, B cells, and CD8^+^ T cells (Figure 10, A and B, and Supplemental Figure 8B). There was a significant increase in *Ccl5* signaling from CD8^+^ T cells directed at *Cd74/C1q/Aif1* macrophages, *Vegfa/Nlrp3* macrophages, and patrolling Mo, indicating enhanced chemotactic recruitment of myeloid cells (Figure 10A and Supplemental Figure 8B). We also observed increased signaling of *Ccl5* from CD8^+^ T cells to B cells, in conjunction with outgoing *Thbs1*, *Ccl6*, *Ccl9*, *Apoe*, *Spp1*, and *Fn1* signals from *Vegfa/Nlrp3* macrophages (Figure 10, A and B, and Supplemental Figure 8B).

### Prevalence of variant MIF promoter alleles in human melanoma.

Functional polymorphisms in the *MIF* gene as defined by a -794 CATT_5-8_ promoter microsatellite (*rs5844572*) occur in the human population. The high-expression -794 CATT_7_ allele is present in approximately 20% of individuals, where it is associated with increased susceptibility to or severity of multiple autoimmune diseases (6). We hypothesized that this naturally occurring variation in *MIF* expression could influence the development of human melanoma. A G-to-C single nucleotide polymorphism (SNP) at position -173 also exists (*rs755622*), with the -173C variant in linkage disequilibrium with the high-expression -794 CATT_7_ *MIF* allele (24). Notably, neither *MIF* promoter variant is represented in currently employed GWAS chips. Due to locus heterogeneity at the -794 CATT_5-8_ microsatellite, genetic association studies in small cohort studies may be significant at the -173 G/C but not at the -794 CATT_5-8_ site. We analyzed the prevalence of the -173 G/C SNP in a retrospective case-control study comprising 182 patients with melanoma from Yale University and 1,575 age- and sex-matched healthy controls. Analysis of -173 G/C genotype frequencies revealed that the high-expression C/C genotype was 2.6 times more frequent in patients compared with controls (*P* < 0.000077; Table 1), suggesting that high genotypic MIF expression may confer risk for melanoma development.

## Discussion

Overcoming primary and acquired resistance to ICIs is an area of critical clinical need and the subject of significant research efforts. Cytokine-directed therapies alone or in combination with ICIs are emerging as promising targets. MIF is a tractable target because of its multiple pro-tumorigenic effects and its role in promoting an immunosuppressive TME (25, 26). Moreover, anti-MIF neutralizing antibodies, MIF-directed RNA silencing, or small molecule MIF inhibitors have been tested in vitro and in preclinical tumor models with notable responses, but studies into the mechanisms of activity remain limited (27–29). Early clinical testing of anti-MIF has shown only modest responses as a single agent, but we and others show evidence for synergism with existing ICIs (10, 13, 30).

We evaluated whether anti-MIF could enhance the therapeutic efficacy of anti–PD-1 in established mouse models and assessed the underlying molecular mechanisms of response. Mice with YUMMER1.7 melanomas and MC38 colorectal carcinomas treated with anti–PD-1/anti-MIF had trends toward slower tumor growth, more complete tumor regression, and prolonged survival when compared with those treated with monotherapy (anti–PD-1 or anti-MIF alone) or isotype control. Treated mice also demonstrated robust and prolonged antitumor memory responses. Tumor histology showed trends toward increased tumor necrosis, immune infiltration, and decreased tumor vascularity after dual inhibition.

Implantation of YUMMER1.7 tumors into MIF-KO mice or mice with a low-expression human *MIF* allele (CATT_5_) showed that decreased host MIF was associated with decreased tumor growth. Once tumors began to grow, the growth rate was similar, which raises questions related to stromal MIF effects on delaying early tumor survival, establishment, or growth. Others have shown similar delays in tumor growth with loss of host MIF expression, potentially due to alterations in macrophage populations and angiogenesis (31–33). Anti–PD-1 treatment of MIF-KO or CATT_5_ mice further enhanced tumor responses, underscoring the additive responses of MIF and PD-1 targeting. Additional studies are needed to fully understand early host-tumor contributions of MIF signaling and the benefit of adding anti–PD-1 therapy in this context. Although not directly compared, there was increased regression of tumors in MIF-KO compared with CATT_5_ mice treated with anti–PD-1. We hypothesize that MIF-KO mice have complete absence of MIF in host cells, whereas CATT_5_ mice have decreased MIF. Low levels of MIF in the host may partially explain the decrease in complete antitumor rejection in CATT_5_ mice. These results are consistent with prior reports of dose-dependent MIF responses in tumor growth (34). Further, no significant differences were observed between parental YUMMER1.7 diploid tumors and YUMMER1.7 diploid *Mif*^–/–^ tumors, likely due to compensatory mechanisms or to continued MIF presence from the host environment. We hypothesize that tumor cells may adapt by upregulating alternative pathways, such as D-dopachrome tautomerase (DDT, a.k.a. MIF-2), with host MIF influencing initial tumor establishment and tumor-secreted MIF being sufficient to support subsequent growth and progression.

As previous studies suggested that MIF can promote immune cell polarization or differentiation toward phenotypes that support an immunosuppressive TME (32), we hypothesized that pharmacologic MIF inhibition could act on the TME to augment antitumor immune responsiveness. Anti-MIF treatment did indeed enhance circulating cytokine profiles consistent with T cell stimulation, T_H_1 differentiation, and macrophage activation. Activated T_H_1 cells are known to be critical to the induction and maintenance of antitumor cytotoxic T lymphocyte (CTL) responses (35), and anti-MIF has been reported to increase CTL responses and IFN-γ expression in an OVA-transfected EL-4 tumor model (36). We found that anti-MIF led to decreases in T_H_2-associated cytokines, which is consistent with the literature. However, the T_H_2 cytokine responses with dual anti–PD-1/anti-MIF therapy were mixed and likely reflect complex systemic signaling in tumor-bearing animals. Further studies are required to determine whether MIF inhibition results in a shift from T_H_2 to a T_H_1 response. Previous studies also support that tumor regression is associated with a shift from a T_H_2 to a T_H_1 response in the tumor-draining lymph nodes and spleen that involves epitope-specific, long-lived memory T cells as well as the presence of M1 macrophages (37). It also has been reported that MIF secretion is enhanced by T_H_2 cells but not T_H_1 cells and that the absence of MIF expression in vitro enhances immunogenic cell death (38). This antitumor immune response was characterized by augmented DC maturation and an elevation in IFN-γ–producing intratumor T cells in the 4T1 breast cancer model (39). Collectively, these findings suggest that MIF has pro-tumorigenic effects by downregulating T cell responses, while MIF neutralization enhances T cell lymphocyte homing into tumors and promotes CTL activity.

DCs play critical roles in antigen processing and presentation, with cDC1s being particularly efficient at initiating CD8^+^ T cell responses in multiple cancers (40). We found an increase in cDC1s and a decrease in TAMs after 2 doses of dual therapy. These data suggest that the combination of PD-1/MIF inhibition also augments innate tumor immunity.

We used scRNA-Seq to identify additional intratumor immune subsets. We identified an increase in both *Cd74/C1q/Aif1*-expressing macrophage and DC populations, with higher expression of antigen processing and presentation genes with anti-MIF–containing therapies. *AIF1* expression is positively correlated with immune infiltration in gliomas, esophageal cancers, and medulloblastomas (41–43). High *AIF1* levels are associated with improved patient survival, supporting a protective role for this macrophage population (41).

Furthermore, the *Cd74/C1q/Aif1* macrophage population in our model had increased activity of the transcription factors *Rfxap*, *Rfxank*, *Rfx5*, and *Ciita*, which are key regulators of MHC class II and antigen presentation. The RFXANK, RFX5, and RFXAP proteins form 3 subunits of the ubiquitously expressed RFX complex, which binds directly to the promoters of all MHC class II genes and associates with other pleiotropic factors to form the MHC class II enhanceosome. *Rfx5* increases the surface expression of HLA-DR molecules, promoting macrophage-dependent expansion of antigen-specific T cells (20). Additionally, there was increased activity of *Npm1* and *Atf5*, 2 transcription factors considered to play crucial roles in monocyte-to-macrophage differentiation through the NF-κB activation pathway (22, 23). We hypothesize that anti–PD-1/anti-MIF therapy may promote monocyte-to-macrophage differentiation and thereby enhance expansion of the antigen-activated macrophage population expressing *Cd74/C1q/Aif1* to augment antitumor immunity.

*Cd74/C1q/Aif1*-expressing macrophages also are characterized by higher expression of the complement components *C1qa* and *C1qb*, which have been positively correlated with immune cell infiltration, apoptosis, and improved survival in previous studies of melanoma (44). The upregulation of these genes within the YUMMER1.7 TME by anti-MIF treatment supports the enhanced antitumor immune response of these macrophages. The observed increase in the expression of MIF-associated and antiapoptotic genes in the anti-MIF and anti–PD-1/anti-MIF treatment groups likely reflects the action of supervening stimuli and a compensatory response in the face of MIF neutralization (45).

Of note, while the *Cd74/C1q/Aif1* macrophage subset expands following anti-MIF treatment, its abundance is not further increased by the addition of anti–PD-1. This likely reflects the limited direct effect of PD-1 blockade on myeloid proliferation or recruitment, consistent with its primary function to enhance T cell responses by lifting inhibitory signals on activated T cells (46). However, dual treatment markedly altered the functional state of these macrophages, enhancing *Ccl* signaling in CD8^+^ T cells and *Vegfa/Nlrp3* macrophages. *Cxcl* signaling also was strengthened in *Cd74/C1q/Aif1* macrophages and B cells. These improvements in *Ccl* and *Cxcl* signaling may facilitate the recruitment and activation of immune cells within the TME. Conversely, YUMMER1.7 tumor cells showed a marked reduction in *Ccl*, *Cxcl*, *Thbs*, *Visfatin*, and *Laminin* signaling after anti–PD-1/anti-MIF treatment, indicating a disruption of key pathways involved in melanoma invasion, metastasis, and immune evasion (47–49). Additional signaling changes, including increased *Cd96* activity in CD8^+^ T cells (supporting effector function) and enhanced *Notch* signaling in B cells (important for maturation and antibody production), suggest a broad reprogramming of immune cell crosstalk.

Notably, *Cd80*, an M1 macrophage marker, exhibited heightened signaling from *Vegfa/Nlrp3* macrophages, potentially enhancing antigen presentation. Similarly, increased *Cd80* signaling in *Cd74/C1q/Aif1* macrophages and CD8^+^ T cells suggest a pro-inflammatory TME. The presence of *Cd80* in CD8^+^ T cells has been linked to enhanced cytotoxic potential, possibly contributing to antitumor immunity (50). We and others have noted a direct correlation between immune infiltration, melanoma responses, and intratumor CD74 levels (26). CD74 is known to be present on a wide range of immune cells, including professional antigen-presenting cells, T cells, and B cells. Further studies are needed to understand CD74 regulation in this context, particularly its increased expression with anti-MIF treatment.

Furthermore, our analysis of specific cell interactions revealed a more interconnected and dynamic immune cell network within the TME that was enhanced by anti–PD-1/anti-MIF treatment when compared with anti–PD-1 monotherapy. This increased interconnectivity suggests a more integrated and complex immune landscape within the TME that aligns with the known immunomodulatory effects of MIF inhibition in promoting a more immunogenic TME (13).

Taken together, these findings support a model in which MIF blockade, particularly in combination with PD-1 inhibition, reprograms the macrophage compartment, shifting from pro-tumorigenic TAMs toward a heterogeneous population enriched for antigen presentation and immune recruitment functions. This functional reprogramming enhances cross-talk with effector T cells and other immune subsets, thereby amplifying antitumor immunity.

Given the prevalence of natural genetic variation in human *MIF* expression and prior studies correlating high genotypic *MIF* alleles and immune infiltration in patient tumors (51), we also analyzed a cohort of patients with melanoma for *MIF* alleles and clinical presentation. We found that the high-expression (-173C/C) *MIF* allele occurred 2.6 times more frequently among patients than healthy controls. Further studies are warranted to confirm this finding in additional cohorts and to define how intrinsic MIF expression may influence melanoma development, progression, or treatment response. This analysis also does not factor in epigenetic regulators of MIF expression or expression of the MIF homolog DDT (or MIF-2), which also engages the cognate MIF receptor CD74 and is expressed in many tumor types, including melanoma (25). Mouse modeling also supported this human genetic finding, as mice genetically deficient in *Mif* or engineered to express a low-expression human *MIF* allele showed delayed tumor growth when compared with their WT or high-expression human *MIF* allele counterparts.

These data support ongoing efforts to therapeutically block MIF signaling in cancer. Therapeutic monoclonal antibodies targeting MIF (imalumab) and its canonical receptor CD74 (milatuzumab) have been developed, with milatuzumab achieving orphan drug designation from the FDA for the treatment of multiple myeloma (52). Imalumab was tested in a phase I trial for patients with advanced solid tumors, with best overall response being stable disease in 33.3% of patients with heavily pretreated cancers (30). Ongoing preclinical studies also are evaluating the role in cancer of DDT (25). Taken together, our results support the hypothesis that the combination of anti-MIF with anti–PD-1 has the potential to stimulate robust antitumor immunity by targeting distinct molecular pathways not achieved by PD-1 blockade alone.

## Methods

### Sex as a biological variable.

The YUMMER1.7 tumor line was derived from a male mouse and is known to be spontaneously rejected when implanted into female mice because of sex-specific immune responses. To avoid treatment-independent effects on tumor growth, and in consultation with Marcus Bosenberg at Yale Cancer Center, we limited experiments to male mice.

### In vivo mouse studies.

C57BL/6J mice (Jackson Laboratory) were housed at Yale University under standard conditions. *Mif*^–/–^ and humanized *MIF*^CATT5^ and *MIF*^CATT7^ mice have been described previously (17, 53). Animals were 7–8 weeks old at the time of the experiments, and our study exclusively utilized male mice, as the disease model employed has been validated only in males. YUMMER1.7 cells, YUMMER1.7 diploid cells (H2B-GFP mouse melanoma cell line), and MC38 (colon adenocarcinoma cells) were obtained from Marcus Bosenberg (Yale Cancer Center). YUMMER1.7 diploid *Mif^–/–^* cells were successfully generated by nucleofection with custom sgRNA and Cas9, following standard protocols. Single-colony isolation was performed, and the knockout was confirmed through PCR, sequencing, and protein validation (Supplemental Methods; Supplemental Figure 3, A and B). Cells were maintained in culture with DMEM supplemented with 10% FBS, 1% nonessential amino acids, and 1% antibiotic-antimycotic (Gibco). Cells were left undisturbed for 3 days prior to use and harvested at 60%–85% confluence. After suspension in PBS, 300,000 cells (YUMMER1.7 and MC38) or 500,000 cells (YUMMER1.7 diploid and YUMMER1.7 diploid *Mif*^–/–^) were subcutaneously injected in the flanks of mice. Tumor growth was measured 2 times per week, with tumor volumes calculated as (0.5233) (length) (width) (height).

After 7 days, mice with palpable YUMMER1.7 or MC38 tumors (40 mm^3^) were randomized and treated by intraperitoneal injection with the following: anti–PD-1 (CD279, BioXCell, clone RMP1-14, catalog BE0146) at 10 mg/kg, anti-MIF (clone IIID.9 at 20 mg/kg) (18, 54), and the combination anti–PD-1/anti-MIF or isotype control (IgG_1_) at equivalent dosages. Treatment was stopped on day 31, and mice were sacrificed when tumors reached 1,000 mm^3^. To evaluate antitumor memory responses after day 70, mice with complete YUMMER1.7 regression were rechallenged with 500,000 tumor cells via subcutaneous flank injection and then with 100,000 tumor cells via left ventricle cardiac instillation.

### Flow cytometry analyses.

Immunophenotyping by flow cytometry was performed on YUMMER1.7 tumors collected in timed studies after either 2 or 4 doses of treatment. Tumor tissue was digested in RPMI/2% FBS supplemented with 0.1 mg/mL collagenase (Sigma) and 1:1,000 DNase I (Sigma) for 30 minutes at 37°C and then mechanically dissociated. Dissociated tumors were passed through a 70 μM filter and washed in RPMI medium. Multicolored labeling for initial immunophenotyping was performed using previously published antibody panels, staining protocols, and gating strategies (55). Validation of immune populations identified by scRNA-Seq was done by flow cytometry on the same tumor samples. Antibodies and gating strategy can be found in Supplemental Table 1 and Supplemental Figure 6.

### scRNA-Seq analysis.

YUMMER1.7 tumors from a separate cohort of mice were harvested and processed as described above to create a single-cell suspension. The cell suspension was subjected to fluorescence-activated cell sorting for CD45 expression, yielding CD45^+^ (immune cell) and CD45^–^ (tumor cell) populations. After sorting, the cells were recombined to enrich for immune cells using 20% CD45^–^ and 80% CD45^+^ cells to achieve a final population of 100,000 cells. This enriched cell suspension was processed using the Chromium 5′ Single-Cell Gene Expression system (10x Genomics) to generate scRNA-Seq libraries following the manufacturer’s protocol. Further analysis details, including cell-cell communication, can be found in the Supplemental Methods.

### Immunohistochemistry.

Representative YUMMER1.7 tumors were stained using H&E or for immune markers via standard protocols (55). Immunostaining was performed using citrate buffer heat-induced epitope retrieval. Primary antibodies against CD3 (clone SP7, Novus Biologicals, 1:100, catalog NB600-1441, RRID:AB_789102), CD8 (clone D4W2Z, Cell Signaling Technology, 1:200, catalog 98941S, RRID:AB_2756376), CD163 (clone EPR19518, Abcam, 1:500, catalog ab182422, RRID:AB_2753196), and CD31 (clone D8V9E, Cell Signaling Technology, 1:100, catalog 77699S, RRID:AB_2722705) were applied overnight at 4°C. Slides then were incubated with the ImmPRESS HRP horse anti-rabbit IgG PLUS Polymer Kit (Vector Laboratories, catalog MP-7801), counterstained, and mounted using Permount (Thermo Fisher Scientific catalog SP15-100). Six random photos were taken from each tissue at 10× original magnification for representative intratumor and peritumor tissue. Positive cells were manually counted or processed using the EBimage R package.

### Cytokine analysis.

Plasma from YUMMER1.7 tumor–bearing mice was sampled at baseline (prior to) and after 2 doses of treatment for evaluation with the Mouse Cytokine/Chemokine 31-Plex Discovery Assay Array (MD31) (Eve Technologies). Samples from 5 animals in each treatment cohort were pooled. Cytokine expression was analyzed as the change from paired baseline values.

### Genotyping.

Human melanomas were obtained by surgical excision, and genomic DNA was isolated to analyze the *MIF* promoter -173 G/C SNP (*rs755622*) following methodologies described previously (18). This polymorphism is not represented in available GWAS analysis chips. Sequences were analyzed using the Staden preGap4 and Gap4 programs.

### Statistics.

FlowJo software (BD Biosciences) and GraphPad Prism (version 8; RRID:SCR_002798) were used for statistical analyses. For pairwise comparisons, either a 2-tailed unpaired Student’s *t* test or Mann-Whitney *U* test was applied, depending on data distribution. Multiple-group comparisons were analyzed using 1- or 2-way ANOVA with Bonferroni post hoc correction, as appropriate. When assumptions of normality or equal variance were violated, nonparametric Kruskal-Wallis or Brown-Forsythe and Welch ANOVA tests were used. Tumor growth was assessed by AUC analysis of mean tumor volumes. Survival analyses were performed using the Kaplan-Meier method with log-rank (Mantel-Cox) tests. Cytokine data were visualized and clustered using the pheatmap package (RRID:SCR_016418) in R Studio (version 1.4.1103; RRID:SCR_000432), with fold-change analysis applied to identify cytokines showing ≥2-fold changes. *P* values less than 0.05 were considered statistically significant.

### Study approval.

The animal experiments were approved by the Yale Office of Animal Research Support Committee (Protocol ID: 2023-10992). All human participants voluntarily took part in the study after providing written informed consent, as approved by the Yale Institutional Review Board.

### Data availability.

The scRNA-Seq dataset and associated metadata are publicly available at NCBI GEO repository, under accession number GSE306115 (https://www.ncbi.nlm.nih.gov/geo/query/acc.cgi?acc=GSE306115). Values for all data points found in graphs are in the Supporting Data Values file.

## Author contributions

TTT conceptualized the project. TTT, GASZ, TC, and CBB conducted the experiments. TTT, LO, GASZ, IK, and RB contributed to data interpretation. The manuscript and figures were prepared by GASZ and TTT and reviewed and finalized by TTT, GASZ, LO, JC, CNV, MP, LL, MEA, SCD, CBB, TC, RPK, LZ, MS, LJ, HMK, IK, and RB.

## Funding support

This work is the result of NIH funding, in whole or in part, and is subject to the NIH Public Access Policy. Through acceptance of this federal funding, the NIH has been given a right to make the work publicly available in PubMed Central.

• NIH National Cancer Institute (NCI) Yale SPORE in Skin Cancer P50 CA121974 (to HMK) and the Yale SPORE in Skin Cancer Career Enhancement Program (to TTT).

• Additional funding provided by grants NIH AR078334 (to RB), NIH T32AR07107 (to LO), R01 CA269286 (to LBJ), and the Kuni Foundation and NIH DE033038 (to RPK).

• Partial support provided by NIH grant NCI T32 CA193200-5 (to JIC).

• The Karin Bain and John Kukral Foundation (to TTT).

## Supplementary Material

Supplemental data

Unedited blot and gel images

Supporting data values

## Figures and Tables

**Table 1:**
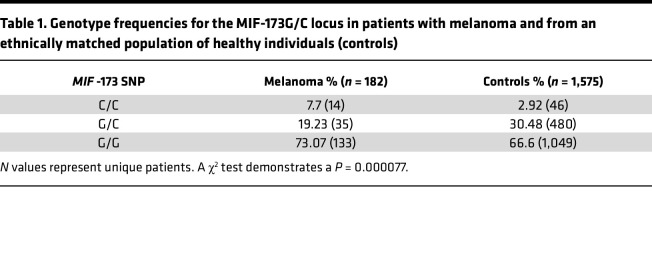
Genotype frequencies for the MIF-173G/C locus in patients with melanoma and from an ethnically matched population of healthy individuals (controls)

## Version 1 10/22/2025

Electronic publication
