# Supplementary material for: Improving immunotherapy responses by dual inhibition of macrophage migration inhibitory factor and PD-1
Source: JCI Insight. 2025 Oct 22;10(20):e191539. doi: 10.1172/jci.insight.191539 (PMC12581657; doi:10.1172/jci.insight.191539)
Supplement: Supplemental data [file jciinsight-10-191539-s017.pdf]

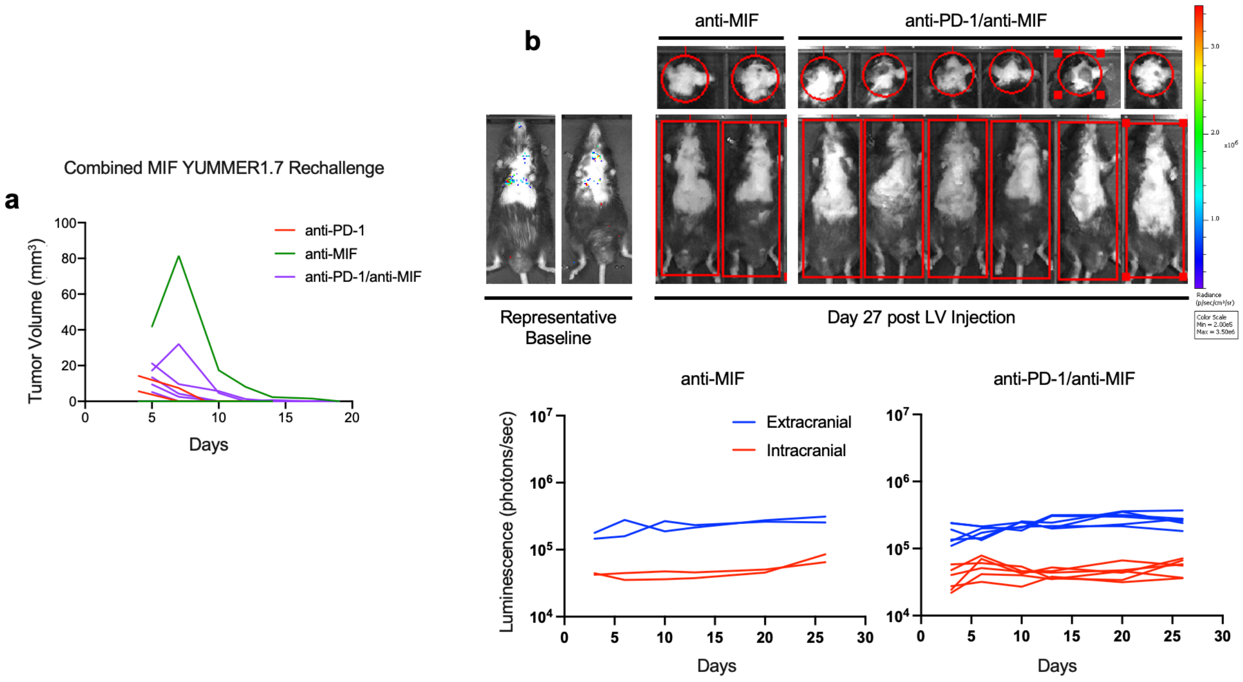

Supplemental Figure 1. Assessment of tumor immune memory in the YUMMER1.7 model. (a) Mice that had previously achieved complete tumor regression from treatment were re-challenged with 500,000 YUMMER1.7 tumor cells via subcutaneous flank injection. Without additional therapy, all re-challenged animals exhibited rapid tumor rejection. (b) To simulate systemic tumor exposure, these animals were subsequently challenged with 100,000 YUMMER1.7 tumor cells via left cardiac ventricle (LV) injection. Immediate imaging confirmed systemic dissemination of tumor cells. All animals demonstrated complete rejection of tumor cells without additional treatment.

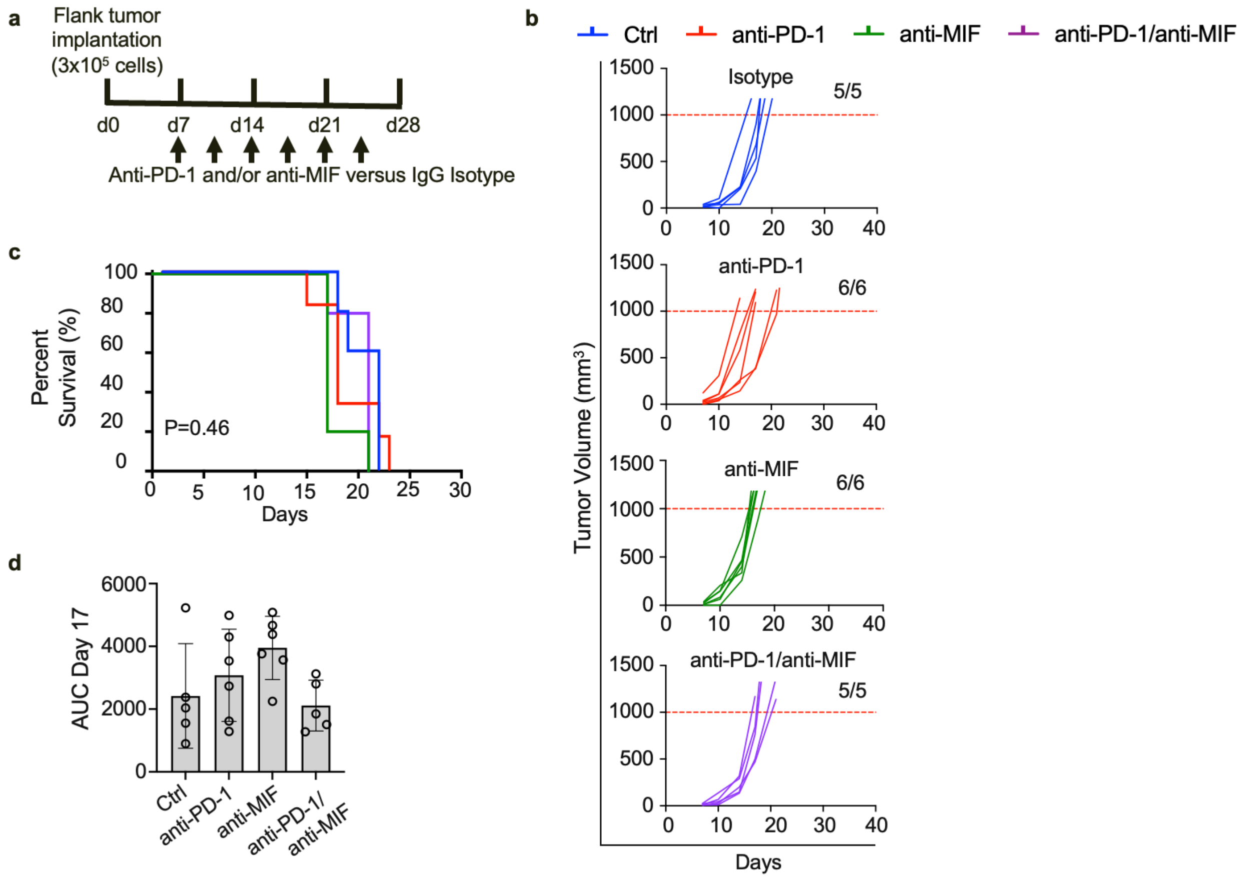

Supplemental Figure 2. Evaluation of treatment efficacy using the B16F10 melanoma tumor model. (a) Seven days after B16F10 tumor implantation, mice were randomized into four treatment groups: anti-PD-1, anti-MIF, combination therapy (anti-PD-1/anti-MIF), and isotype control (Ctrl). (b,c) Tumor volume and overall survival were monitored throughout the study. No significant differences in tumor progression or survival rates were observed among the treatment groups ($p > 0.05$), with tumor endpoint defined as a volume exceeding 1,000 mm³. (d) Tumor area under the curve (AUC) by day 17 showed no differences between the treatment groups.

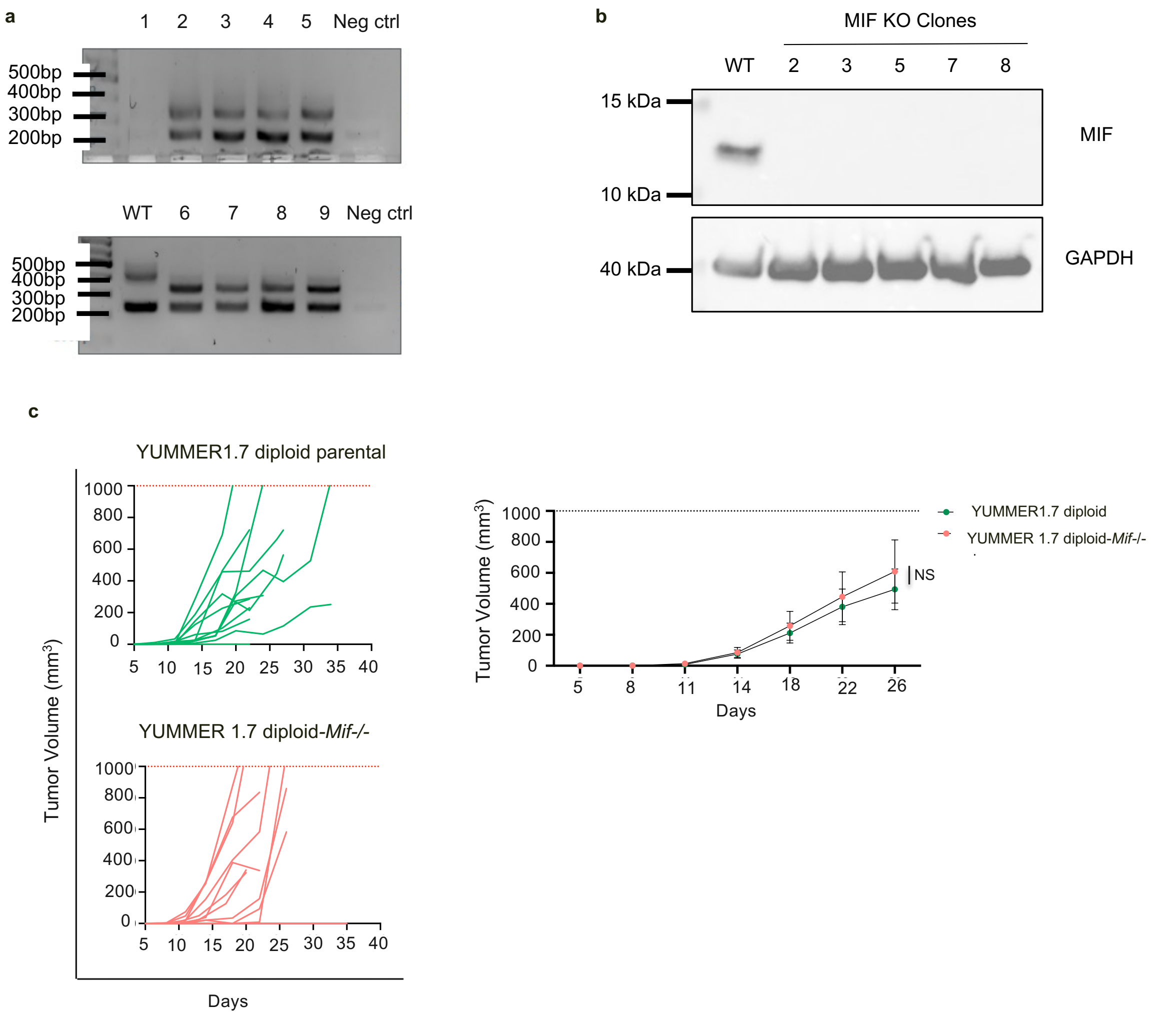

Supplemental Figure 3. CRISPR knockout of *Mif* in YUMMER1.7 diploid melanoma cells does not impact tumor growth in WT mice. (a) PCR analysis of genomic DNA from YUMMER1.7 diploid cells confirmed the presence of the expected band sizes corresponding to wild-type (WT, 450bp), *Mif* knockout (350bp), and *Mif* pseudogenes (~250bp). (b) Western blot analysis verified the complete absence of MIF protein in YUMMER1.7 diploid-*Mif*-/- cells, indicating successful knockout of *Mif*. (c) Tumor growth comparison between YUMMER1.7 diploid-*Mif*-/- and YUMMER1.7 diploid parental tumors implanted in WT mice revealed no significant differences in growth, suggesting that tumor *Mif* loss does not affect tumor growth in this model.

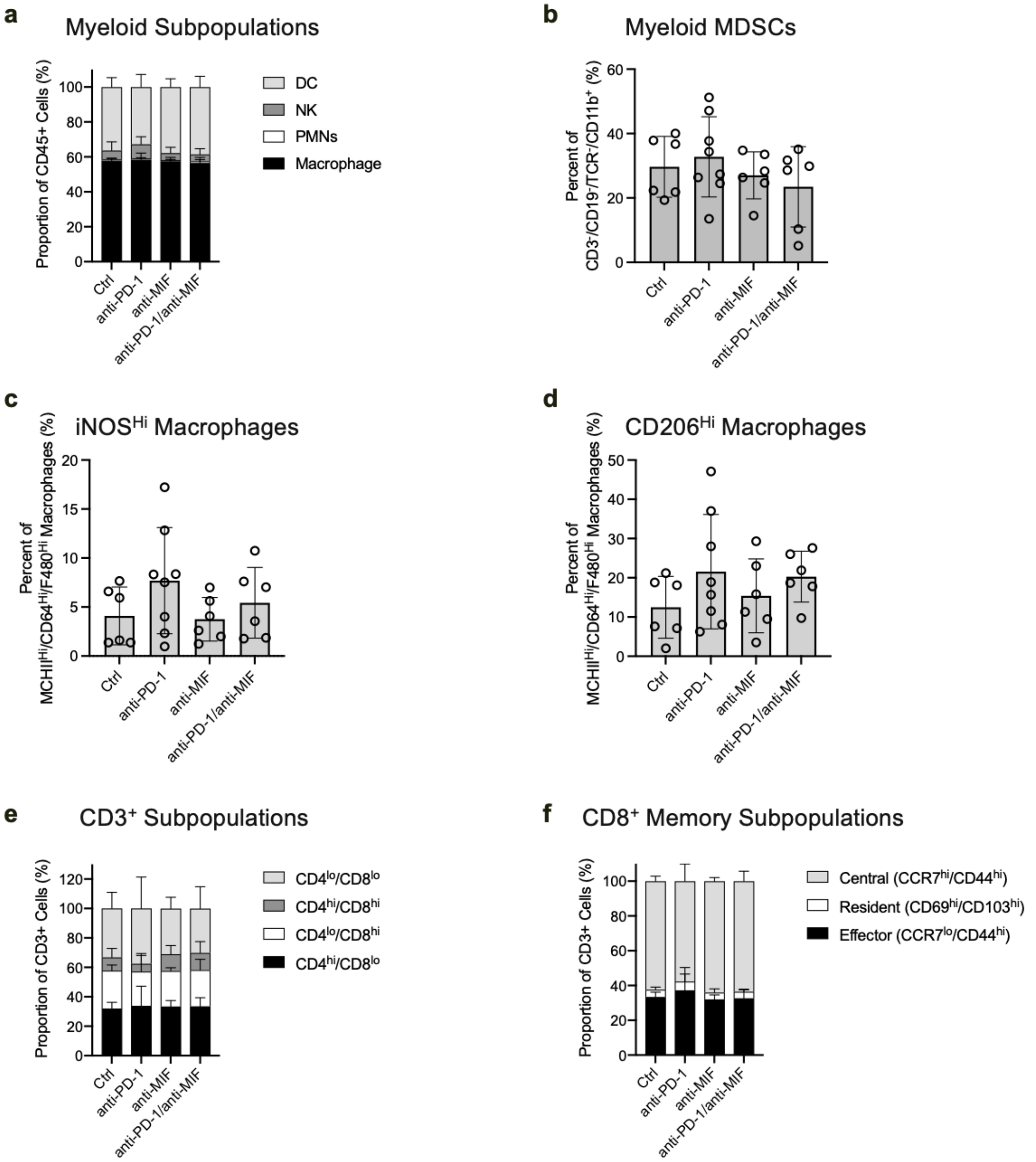

Supplemental Figure 4. Distribution of intratumoral immune cell subtypes analyzed by flow cytometry. Flow cytometry analysis of tumor-infiltrating immune cell populations in YUMMER1.7 tumors after two doses of treatment. No significant differences were observed among treatment groups in the frequencies of (a) myeloid cells, (b) MDSCs, (c) M1-like macrophages, (d) M2-like macrophages, (e) CD3+ T cells, or (f) CD8+ memory T cell subpopulations ($p > 0.05$).

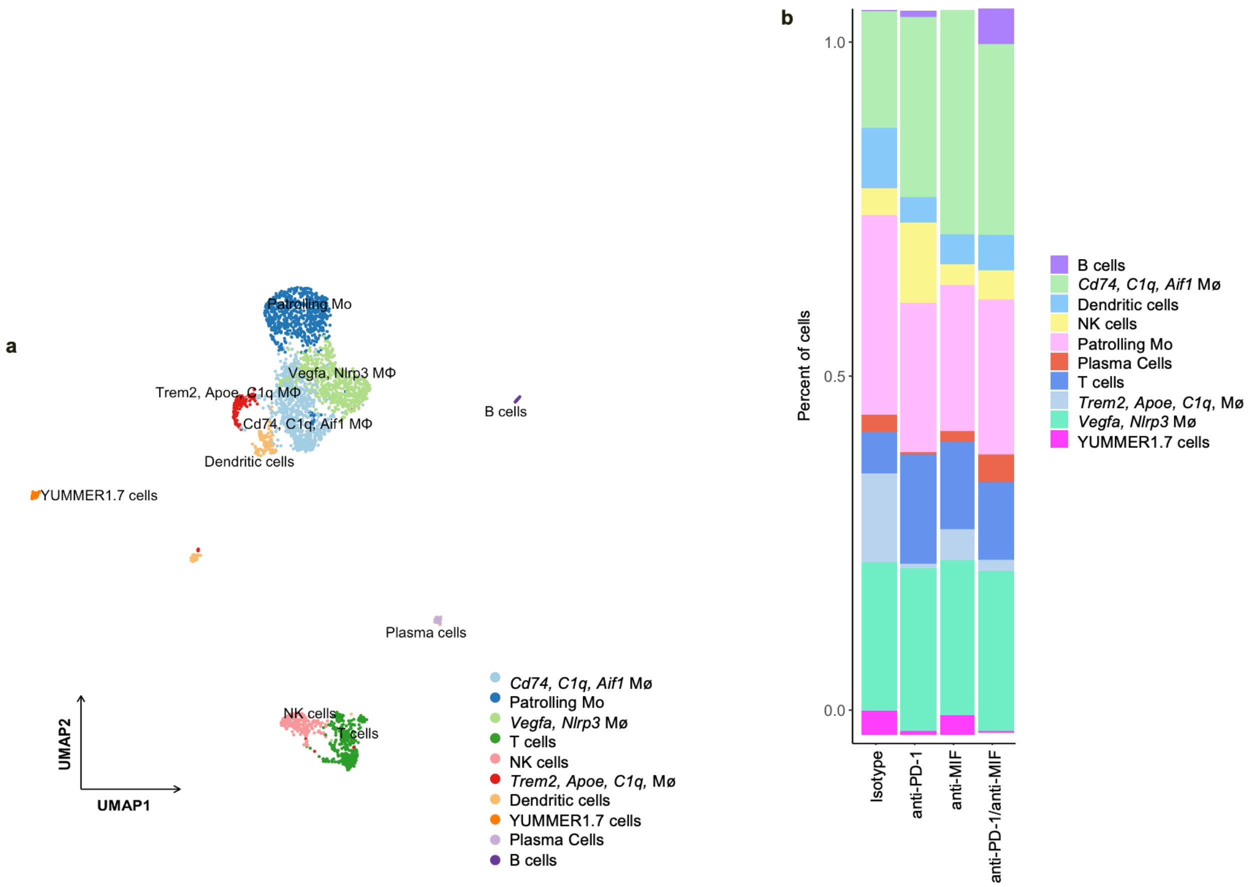

Supplemental Figure 5. Early Expansion of *Cd74/C1q/Aif1*-Expressing Macrophages in the Tumor Microenvironment Following MIF Treatment. (a) YUMMER1.7 melanoma cells were subcutaneously injected into mice, followed by a 1-week treatment (2 doses) course. Tumors were then harvested for scRNA-seq analysis. Unsupervised clustering identified 10 distinct cell populations across different treatment groups, with myeloid cells comprising the majority of immune cells in the TME. (b) Anti-MIF containing groups showed a significant expansion of a macrophage population characterized by *Cd74, C1q,* and *Aif1* expression compared to other treatment groups.

Supplemental Table 1. Flow Antibodies

Color	Antibody	Company	Catalog	Clone	Dilution
BUV395	F4/F80	BD	565614	T45-2342	1:100
AmCyan	Live/Dead	Thermo Fisher	L34965		1:100
BUV737	Cd3	BD	612803	17A2	1:100
BUV737	Cd19	Invitrogen	367-0193-80	1D3	1:50
Pacific Blue	Aif1	CellSignaling	84037S	E4O4W	1:100
AF488	Cd74	Biolegend	151006	CLIP	1:100
PE-Cy5.5	Cd45	Invitrogen	35-0451-80	30-F11	1:50
PE	C1q	BD	569965	RmC7H8	1:100

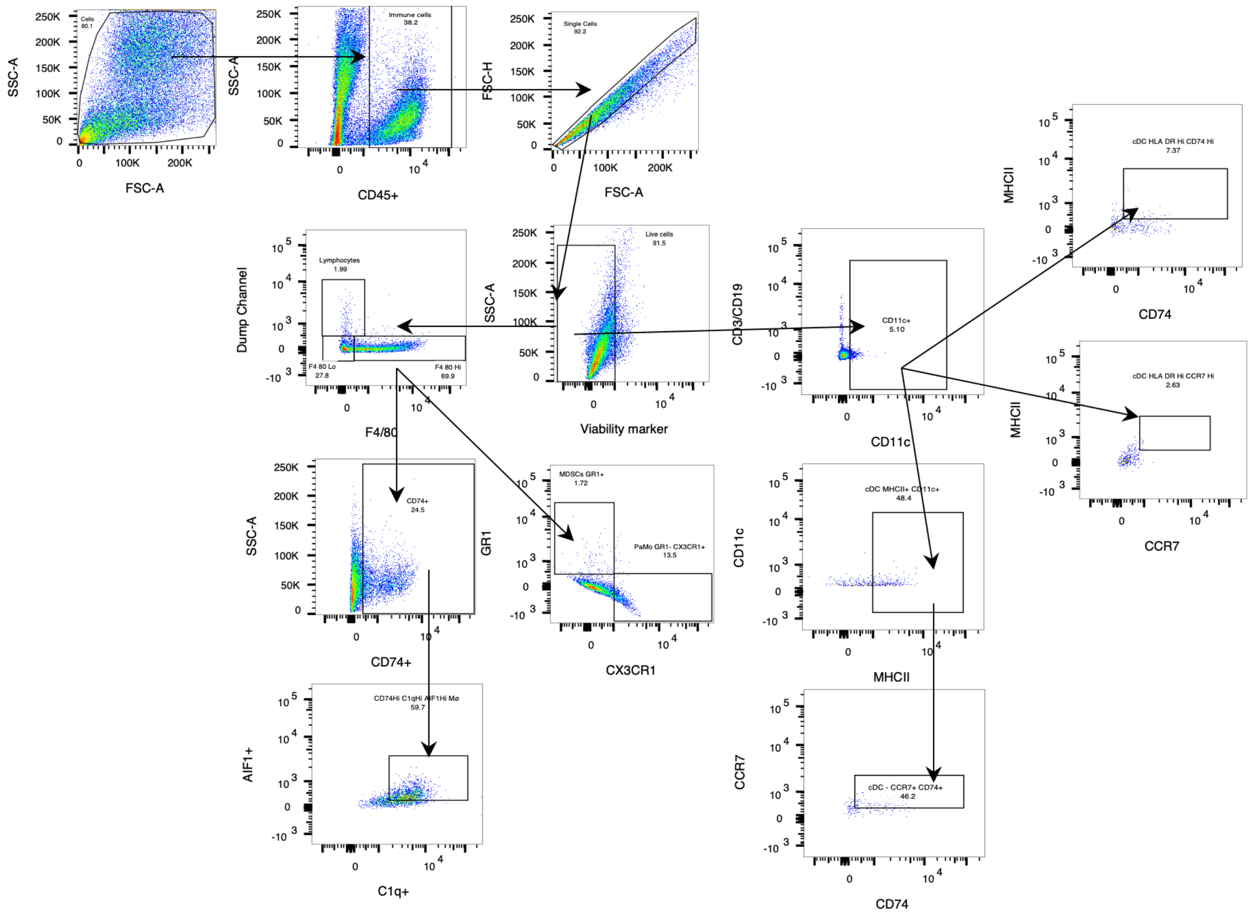

Supplemental Figure 6. Flow Gating Strategy of YUMMER1.7 Tumor Infiltrating Myeloid Populations. Example gating of macrophage and dendritic cells.

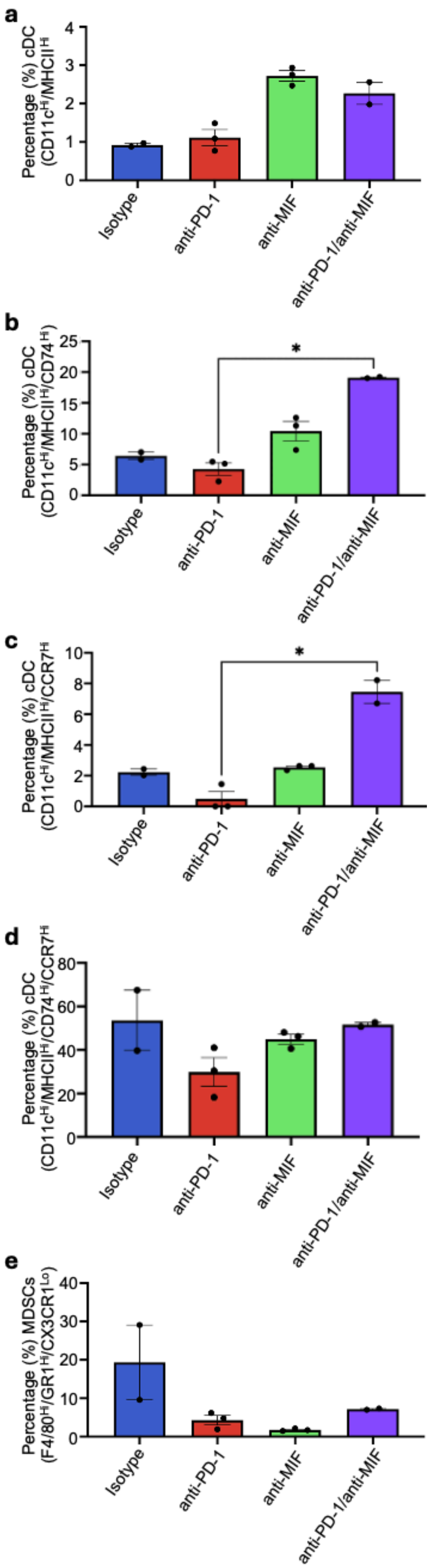

Supplemental Figure 7. Flow Validation of YUMMER1.7 Tumor Infiltrating Populations in the Long Treatment Cohort. (a) Anti-MIF containing therapies showed a non-significant trend towards increased conventional dendritic cells (cDCs). (b) Combined anti-PD-1/anti-MIF treatment resulted in a higher percentage of CD74^{Hi} expressing cDCs compared to anti-PD-1 therapy alone. (c) Combined anti-PD-1/anti-MIF treatment resulted in a higher percentage of CCR7^{Hi} expressing cDCs compared to anti-PD-1 therapy alone. (d) Combined anti-PD-1/anti-MIF treatment did not result in a higher percentage of dual CD74^{Hi}/CCR7^{Hi} cDCs compared to anti-PD-1 therapy alone, consistent with scRNA sequencing results. (e) A non-significant trend towards decreased MDSCs was seen with anti-PD-1 and/or anti-MIF therapy compared to isotype control. Statistical significance is denoted as *$p<0.05$.

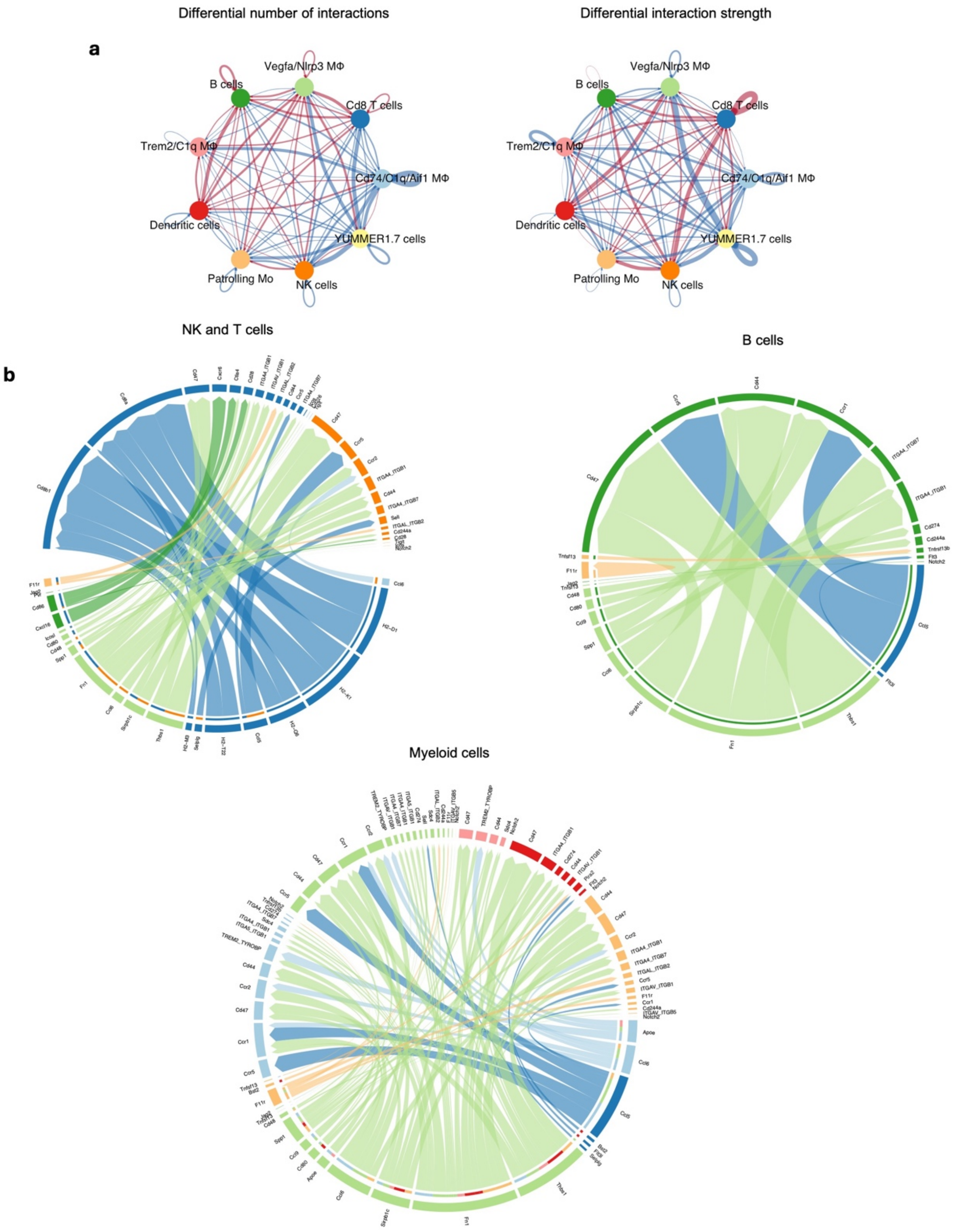

Supplemental Figure 8. Key changes in immune cell interactions and signaling dynamics in the TME upon anti-PD-1/anti-MIF treatment. (a) Network diagrams showing the differential number of interactions (left) and interaction strength (right) among various immune cell types in

the tumor microenvironment (TME) between anti-PD1 and anti-PD1/anti-MIF treatment groups. Red lines indicate increased interactions, while blue lines indicate decreased interactions in the anti-PD1/anti-MIF treatment group compared to the anti-PD1 group. (b) Specific signaling changes in key immune cell types under anti-PD1/anti-MIF compared to anti-PD1 treatments.

Supplemental methods

1. CRISPR/Cas9-Mediated *Mif* Knockout in YUMMER1.7 Diploid cells

Since YUMMER1.7 is tetraploid, deletion of *Mif* was accomplished using a diploid clone of the line. Knockout of *Mif* in YUMMER1.7 diploid cells (YUMMER1.7 diploid-*Mif*-/-) was performed using a CRISPR/Cas9 system and the following sgRNA sequences (Synthego):

5'-GUGUGCCGGCGGGUGGCGGC-3'
3'-UCCUCCCUGCAAACCUGUGC-5´

YUMMER1.7 diploid cells at 70-80% confluency were transfected using the Lonza SF Cell Line nucleofection kit (Cat #V4XC-2032). Briefly, cells were resuspended in SF Cell Line solution and mixed with Cas9/sgRNA complex or control reagents. The nucleofection was carried out using the DJ-110 program on the Lonza Nucleofector following the manufacture's instruction for B16 cells. Post-electroporation, cells were transferred to pre-warmed media, incubated for 10 minutes, and then plated in 24-well plates for subsequent analysis.

Monoclonal YUMMER1.7 diploid-*Mif*-/- cell lines were generated using limiting dilution. Cells were cultured in DMEM supplemented with 10% FBS, 1% antibiotic-antimycotic (A/A), and 1% non-essential amino acids (NEAA), and maintained at 37°C in a humidified incubator with 5% CO_2. The successful knockout of *Mif* was confirmed by PCR, demonstrating the deletion of the target gene segment, and by western blot analysis, confirming the absence of MIF protein expression (Supplemental Fig. 3A,B). Clone 7, validated through these assays, was selected for use in subsequent experiments.

2. Single-cell RNA sequencing

The Cell Ranger(1) (version 7.1.0, 10x Genomics) pipeline was used to process the raw single cell RNA sequencing (scRNA-seq) data including alignment to the mouse reference genome, mm10, transcript quantification and generation of gene-cell matrices. The resulting filtered gene-cell count matrices were further analyzed using the R (version 4.3.2) based Seurat(2) package (version 5.1.0) package, following the standard Seurat analysis pipelines for pre-processing, normalization, scaling, dimension reduction and clustering.

Cells were demultiplexed using hashtag oligo (HTO) enrichment(3), after which doublets and negatives were removed. Cells with greater than 5000 or less than 200 unique features, and cells with greater than 5% mitochondrial counts were filtered out. 1671 cells remained after pre-processing and filtering. Gene expression for each cell was normalized and log transformed. Each feature was scaled to a mean of 0 and by the standard deviation for each feature.

Dimension reduction was performed with Principal Component Analysis (PCA) and Uniform Manifold Approximation and Projection (UMAP)(4,5), using 30 principal components. The cells were embedded in a K-nearest neighbor graph, after which clustering was performed using the Louvain(6) community detection algorithm with a resolution of 0.4.

Differentially expressed genes (DEGs) were identified using DESeq2(7) (version 1.41.2). P values were adjusted using the Benjamini-Hochberg(8) method. The EnhancedVolcano(9) (version 1.20.0) package was used to visualize DEGs. The dittoseq(10) (version 1.14.2) package was utilized to determine cluster proportions across treatment groups and to generate stacked bar plots. Clusters were annotated using DEGs and known canonical markers.

A myeloid cell subset was created for further analysis. Scaling, dimension reduction, clustering with a resolution of 0.6 and differential gene expression analysis was performed as above. Clusters were annotated using DEGs.

Gene Set Enrichment Analysis (GSEA)(11) and Over-Representation Analysis (ORA) were performed using the clusterProfiler(12) (version 4.10.1) package to identify enriched biological processes and pathways. GSEA and ORA were performed using gene sets from Gene Ontology(13,14) Biological Processes (GO BP) and KEGG(15,16) (Kyoto Encyclopedia of Genes and Genomes) databases. For ORA, a log2(fold change) > 0.5 was used as the cut-off for upregulated genes.

Gene sets comprised of *Mif* associated genes and anti-apoptosis genes were created. Module scores for expression of these gene sets as well as the 'KEGG Antigen Processing and Presentation' gene set were calculated for each cluster using Seurat. Kruskal-Wallis testing was performed for statistical comparison of module scores using the ggpubr(17) (version 0.6.0) package.

Cell-cell communication analysis was performed using the CellChat(18) (version 2.1.2) package using the standard pipeline. Cell communication was inferred from putative ligand-receptor interactions. Cell types with fewer than 5 cells per treatment condition were excluded. Comparative and differential ligand-receptor expression analysis was performed between the anti-PD1-MIF and anti-PD1 treatment groups. Transcription factor activity inferential analysis was performed using the python package decoupler (version 1.7.0)(19,20).

References:

1. Zheng GXY, Terry JM, Belgrader P, Ryvkin P, Bent ZW, Wilson R, et al. Massively parallel digital transcriptional profiling of single cells. Nat Commun. 2017 Jan 16;8:14049.

2. Hao Y, Stuart T, Kowalski MH, Choudhary S, Hoffman P, Hartman A, et al. Dictionary learning for integrative, multimodal and scalable single-cell analysis. Nat Biotechnol. 2024 Feb;42(2):293–304.

3. Stoeckius M, Zheng S, Houck-Loomis B, Hao S, Yeung BZ, Mauck WM, et al. Cell Hashing with barcoded antibodies enables multiplexing and doublet detection for single cell genomics. Genome Biol. 2018 Dec;19(1):224.

4. McInnes L, Healy J, Melville J. UMAP: Uniform Manifold Approximation and Projection for Dimension Reduction [Internet]. arXiv; 2018 [cited 2024 Jul 16]. Available from: https://arxiv.org/abs/1802.03426

5. Becht E, McInnes L, Healy J, Dutertre CA, Kwok IWH, Ng LG, et al. Dimensionality reduction for visualizing single-cell data using UMAP. Nat Biotechnol. 2018 Dec 3;

6. Blondel VD, Guillaume JL, Lambiotte R, Lefebvre E. Fast unfolding of communities in large networks. J Stat Mech. 2008 Oct 9;2008(10):P10008.

7. Love MI, Huber W, Anders S. Moderated estimation of fold change and dispersion for RNA-seq data with DESeq2. Genome Biol. 2014;15(12):550.

8. Benjamini Y, Hochberg Y. Controlling the False Discovery Rate: A Practical and Powerful Approach to Multiple Testing. Journal of the Royal Statistical Society: Series B (Methodological). 1995 Jan;57(1):289–300.

9. Kevin Blighe, Sharmila Rana, Myles Lewis. EnhancedVolcano: Publication-ready volcano plots with enhanced colouring and labeling. R package version 1.12.0 [Internet]. Available from: https://github.com/kevinblighe/EnhancedVolcano

10. Bunis DG, Andrews J, Fragiadakis GK, Burt TD, Sirota M. dittoSeq: universal user-friendly single-cell and bulk RNA sequencing visualization toolkit. Bioinformatics. 2021 Apr 1;36(22–23):5535–6.

11. Subramanian A, Tamayo P, Mootha VK, Mukherjee S, Ebert BL, Gillette MA, et al. Gene set enrichment analysis: a knowledge-based approach for interpreting genome-wide expression profiles. Proc Natl Acad Sci U S A. 2005 Oct 25;102(43):15545–50.

12. Wu T, Hu E, Xu S, Chen M, Guo P, Dai Z, et al. clusterProfiler 4.0: A universal enrichment tool for interpreting omics data. Innovation (Camb). 2021 Aug 28;2(3):100141.

13. Ashburner M, Ball CA, Blake JA, Botstein D, Butler H, Cherry JM, et al. Gene Ontology: tool for the unification of biology. Nat Genet. 2000 May;25(1):25–9.

14. The Gene Ontology Consortium, Aleksander SA, Balhoff J, Carbon S, Cherry JM, Drabkin HJ, et al. The Gene Ontology knowledgebase in 2023. Baryshnikova A, editor. GENETICS. 2023 May 4;224(1):iyad031.

15. Kanehisa M. KEGG: Kyoto Encyclopedia of Genes and Genomes. Nucleic Acids Research. 2000 Jan 1;28(1):27–30.

16. Kanehisa M, Furumichi M, Sato Y, Kawashima M, Ishiguro-Watanabe M. KEGG for taxonomy-based analysis of pathways and genomes. Nucleic Acids Research. 2023 Jan 6;51(D1):D587–92.

17. Kassambara A. ggpubr: "ggplot2" Based Publication Ready Plots [Internet]. Available from: https://CRAN.R-project.org/package=ggpubr

18. Jin S, Plikus MV, Nie Q. CellChat for systematic analysis of cell-cell communication from single-cell and spatially resolved transcriptomics [Internet]. 2023 [cited 2024 Jul 16]. Available from: http://biorxiv.org/lookup/doi/10.1101/2023.11.05.565674

19. Badia-i-Mompel P, Vélez Santiago J, Braunger J, Geiss C, Dimitrov D, Müller-Dott S, et al. decoupleR: ensemble of computational methods to infer biological activities from omics data. Kuijjer ML, editor. Bioinformatics Advances. 2022 Jan 10;2(1):vbac016.

20. Türei D, Valdeolivas A, Gul L, Palacio-Escat N, Klein M, Ivanova O, et al. Integrated intra- and intercellular signaling knowledge for multicellular omics analysis. Molecular Systems Biology. 2021 Mar;17(3):e9923.
