# Supplementary material for: Improving immunotherapy responses by dual inhibition of macrophage migration inhibitory factor and PD-1
Source: JCI Insight. 2025 Oct 22;10(20):e191539. doi: 10.1172/jci.insight.191539 (PMC12581657; doi:10.1172/jci.insight.191539)
Supplement: Unedited blot and gel images [file jciinsight-10-191539-s018.pdf]

Supplemental Figure 3. CRISPR knockout of *Mif* in YUMMER1.7 diploid melanoma cells does not impact tumor growth in WT mice

(a) PCR analysis of genomic DNA from YUMMER1.7 diploid cells confirmed the presence of the expected band sizes corresponding to wild-type (WT, 450bp), *Mif* knockout (350bp), and *Mif* pseudogenes (~250bp).

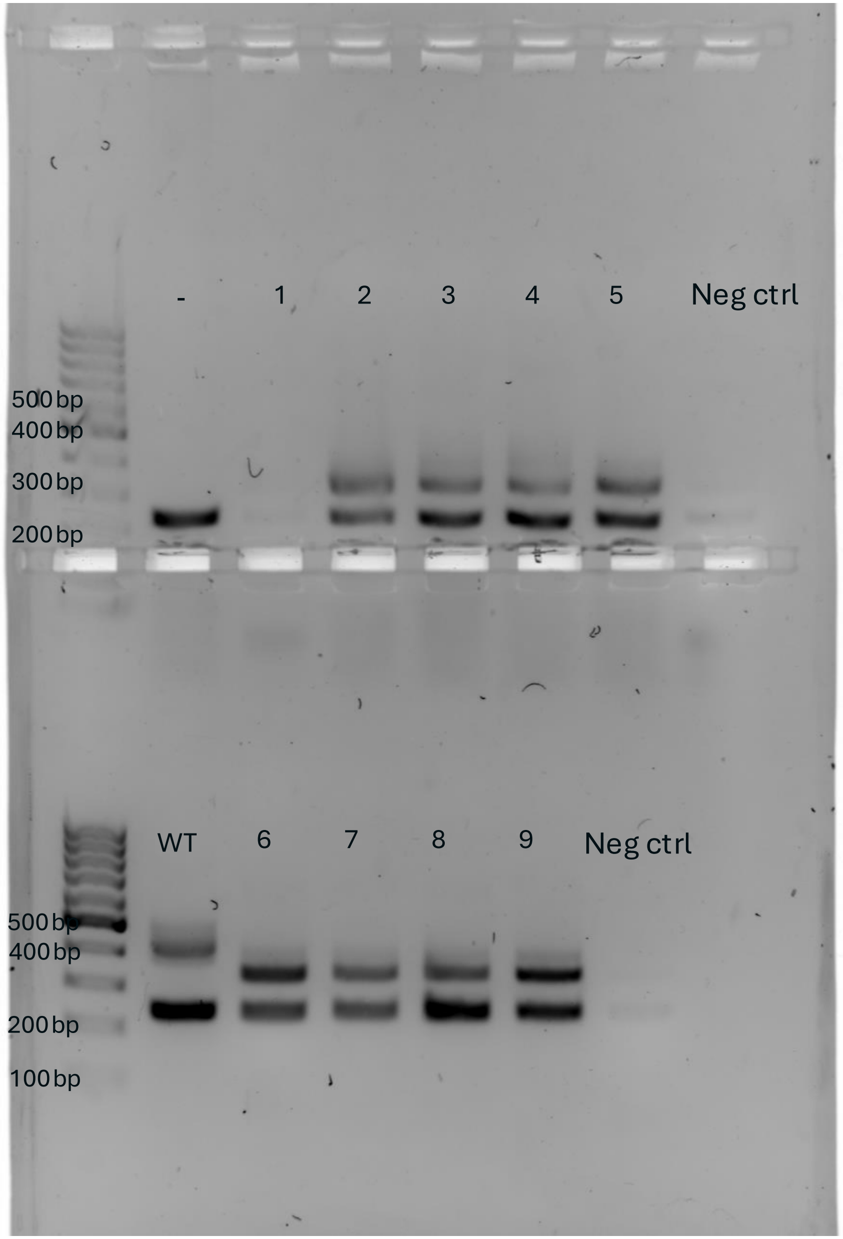

(b) Western blot analysis verified the complete absence of MIF protein in YUMMER1.7 diploid-*Mif*-/- cells, indicating successful knockout of *Mif*.

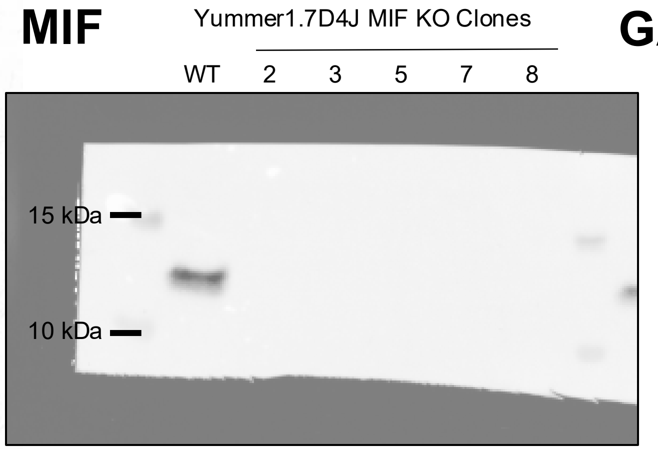

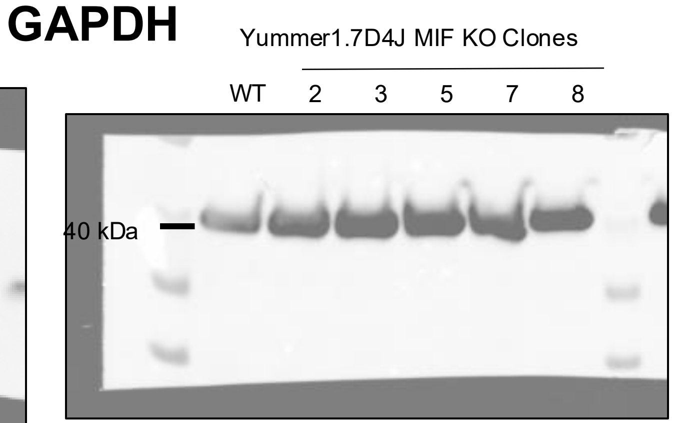

Antibody	Catalog	Dilution
Rabbit anti-MIF (E7T1W) mAb	Cell Signaling, Cat#87501	1:10,000 dilution
Anti-rabbit HRP	Cell Signaling, Cat #7074S	1:1,000 dilution

Antibody	Catalog	Dilution
Mouse GAPDH (D4C6R) mAb	Cell Signaling, Cat ##97166	1:10,000 dilution
Anti-mouse HRP	Cell Signaling, Cat #7076S	1:1,000 dilution

Figure. 1. Anti-PD-1/anti-MIF improves survival and tumor responses.

(i) MIF levels were detected in cell lysate of YUMMER1.7 and MC38 cells

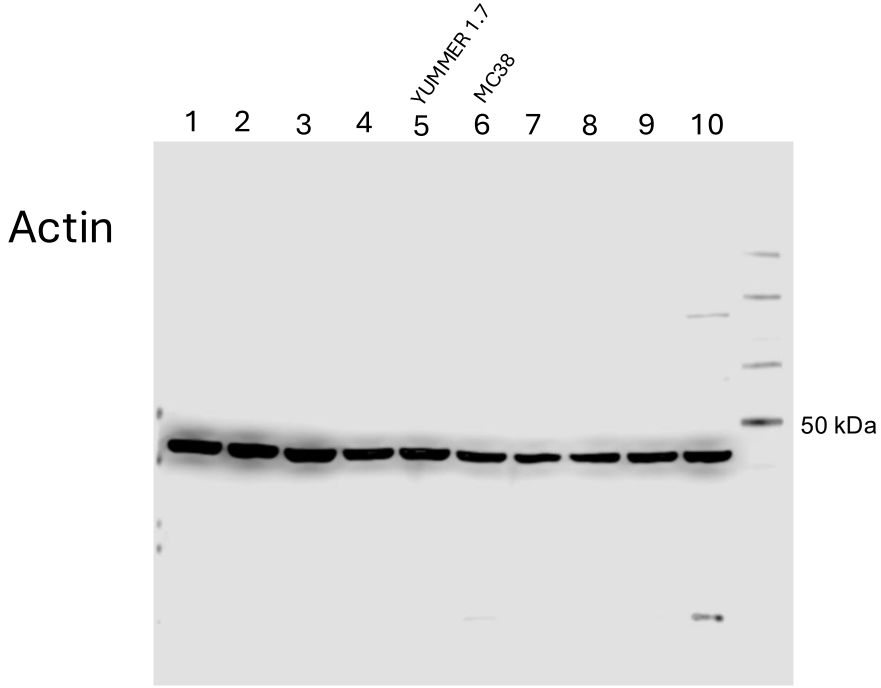

Antibody	Catalog	Dilution
Mouse anti-actin mAb	Cell Signaling, Cat #3700S	1:10,000 dilution
Anti-mouse HRP	Cell Signaling, Cat #7076S	1:1,000 dilution

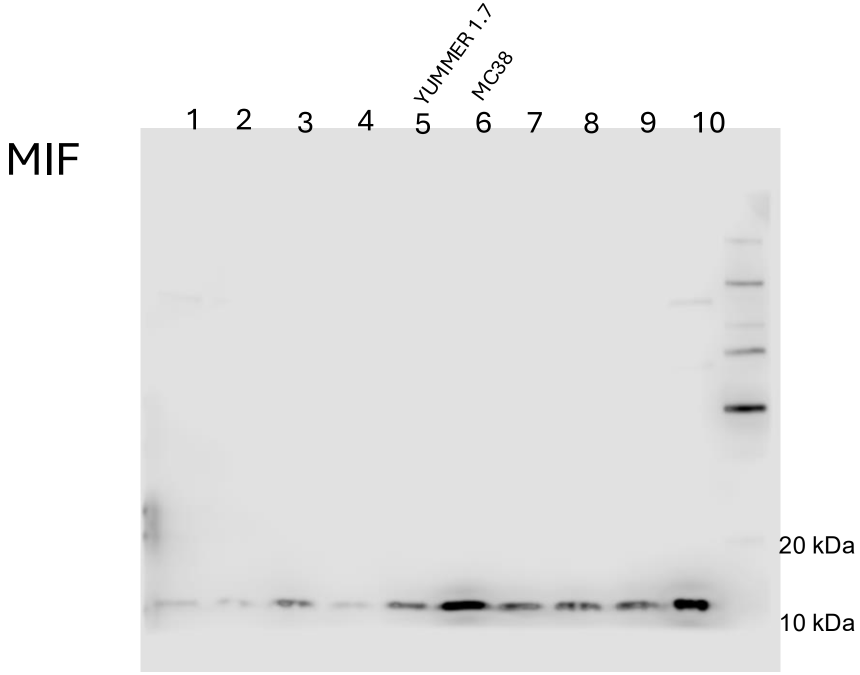

Antibody	Catalog	Dilution
Rabbit anti-MIF (E7T1W) mAb	Cell Signaling, Cat#87501	1:10,000 dilution
Anti-rabbit HRP	Cell Signaling, Cat #7074S	1:1,000 dilution
